# Peas (*Pisum sativum* subsp. *arvense* Asch) and Beans (*Vicia faba* var. *minor*) as Source of Quality Plant Proteins

**DOI:** 10.3390/molecules30092009

**Published:** 2025-04-30

**Authors:** Abebaw Tiruneh, Paweł Ptaszek, Daniel Żmudziński, Tomasz Tarko

**Affiliations:** 1 Department of Engineering and Machinery in Food Industry, Faculty of Food Technology, University of Agriculture in Krakow, Balicka 122, 30-149 Krakow, Poland; abebaw.tiruneh@student.urk.edu.pl (A.T.); daniel.zmudzinski@urk.edu.pl (D.Ż.); 2 Faculty of Chemical and Food Engineering, Bahir Dar Institute of Technology, Bahir Dar University, Bahir Dar P.O. Box 26, Ethiopia; 3 Center for Innovation and Research on Prohealthy and Safe Food, University of Agriculture in Krakow, ul. Balicka 104, 30-149 Krakow, Poland; 4 Department of Fermentation Technology and Microbiology, Faculty of Food Technology, University of Agriculture in Krakow, Balicka 122, 30-149 Krakow, Poland

**Keywords:** plant protein, field bean protein, field pea protein, extraction, chemical composition, functionality, structural modification, food applications, nutrition properties

## Abstract

The demand for plant-based proteins has grown significantly due to their sustainability and lower environmental impact compared to animal proteins. Shifting from animal-based to plant-based diets, particularly those incorporating protein-rich legumes like beans and peas, can substantially reduce the climate footprint of food production. Underutilized legumes, which are often critical in resource-poor regions, hold immense potential for enhancing food security, nutrition, and agricultural development. Despite their importance, information about these legumes remains limited and region-specific. The shift towards plant proteins is further driven by the growing popularity of vegetarian and vegan diets, alongside mounting concerns over the environmental impacts of livestock farming. Consequently, plant proteins are increasingly favored over their animal-based counterparts in the food industry. Scientists are now exploring novel plant protein sources and developing superior-quality proteins with enhanced functional and nutritional characteristics using cutting-edge technologies. While traditional plant protein sources like wheat and soy present challenges such as allergenicity, pulses like peas, beans, chickpeas, and lentils are gaining prominence due to their agronomic and nutritional advantages. It is anticipated that ongoing research will address the existing knowledge gaps regarding the nutritional and health benefits of fodder seeds such as field bean and field pea seeds, broadening their application across diverse food industries. In this context, the present review focuses on the potential of field bean and field pea as valuable sources of food and functional ingredients. Despite their benefits, current knowledge about these crops is limited to specific geographic areas where they hold cultural or local significance.

## 1. Introduction

The food market and industry have changed recently due to the increased demand and investments made in new and alternative protein sources [1,2,3,4]. There is a growing interest in the development of plant-based foods due to a number of factors, including the growing global population and protein demand, social awareness of the negative environmental impacts of animal-based food production, fair food production practices, affordability, ethical and health concerns, and a desire for high-quality nutritional sources. It is now evident that a dietary shift away from animal protein is necessary for sustainability and food security [1,2,3,5,6,7,8,9,10,11,12]. By 2050, the global population is projected to reach 10 billion, with agriculture contributing up to 30% of greenhouse gas emissions [1,2,10,13]. Excessive consumption of animal-based protein is linked to non-communicable diseases like obesity, type II diabetes, heart disease, and cancer [1,3]. To address this, shifting towards sustainable, animal-free protein sources is essential [14,15,16]. It is increasingly acknowledged that producing plant crops high in protein for animal feed is frequently less sustainable and effective than consuming plant proteins directly by people. This encourages the development and exploitation of additional plant protein sources, such as pulses [9,17]. Pulses, along with cereals, pseudocereals, and nuts, are increasingly used in the food industry as sustainable protein sources [2,3,18,19,20,21]. In this context, pulses dry edible seeds of leguminous crops like peas, faba beans, lentils, chickpeas, lupins and cowpeas are rich in protein, carbohydrates, dietary fibre, fats, vitamins, and minerals [5,9,11,14]. They promote circulatory health and may help to prevent neurodegenerative diseases [14,16], cancer, and cardiovascular conditions [10]. These qualities make pulses an excellent substitute for animal protein, especially given their wide acceptance and bioaccessibility [5]. Pulses also provide valuable functional properties, including solubility, emulsification, and foaming, making them versatile in food production [14,15,16,22]. From an environmental perspective, pulses offer significant benefits; they provide and mobilise nitrogen in the soil, require minimal fertilisers, have a low carbon footprint, are water-efficient, and are cost-effective to produce [14,15,23]. Furthermore, grain legumes, beyond its nutritional value, is also a rich source of bioactive compounds that have reported health-enhancing properties. These include phenolic compounds, resistant starch, dietary fibres, non-protein amino acids (L-DOPA, GABA), and, foremost, bioactive peptides [11,21,24]. These compounds have countless useful properties like anti-inflammatory, antibacterial, antioxidant, anticarcinogenic, and antidiabetic effects [25,26]. This makes them a sustainable and nutritious protein source for the future [11].

The pulses-based protein market is primarily dominated by soy [2]. However, with rising demand, there is a need to diversify and introduce new sources, such as other pulse crops [21]. One such crop is the fava bean (*Vicia faba* L.), also known as the field bean, horse bean, or broad bean, and it is commonly cultivated as a food and feed for animal usage [27,28,29,30,31,32]. This cool-season annual pulse has been cultivated for thousands of years across Asia, Africa, Latin America, and the Mediterranean, yet it remains under-utilized in Western countries. Fava beans are adaptable to various climates, including colder regions [21,22,23,33]. There are different varieties based on their seed size: *V. faba* var. *major* with large broad flat seeds (mainly grown in China and South Mediterranean countries), *V. faba* var. *equine* with medium-sized seeds (mainly grown in North Africa and Middle Eastern countries), and *V. faba* var. *minor* with small and rounded seeds (generally grown in Ethiopia and North Europe) [16,27,30]. Faba bean seed size is an important trait in determining market and consumption forms. *V. faba* var. *major* is commonly used for food (either as fresh green vegetable or dry seeds). *V. faba* var. *minor* and var. *equina* are mostly used for animal feed [30,32].

**Figure 1:**
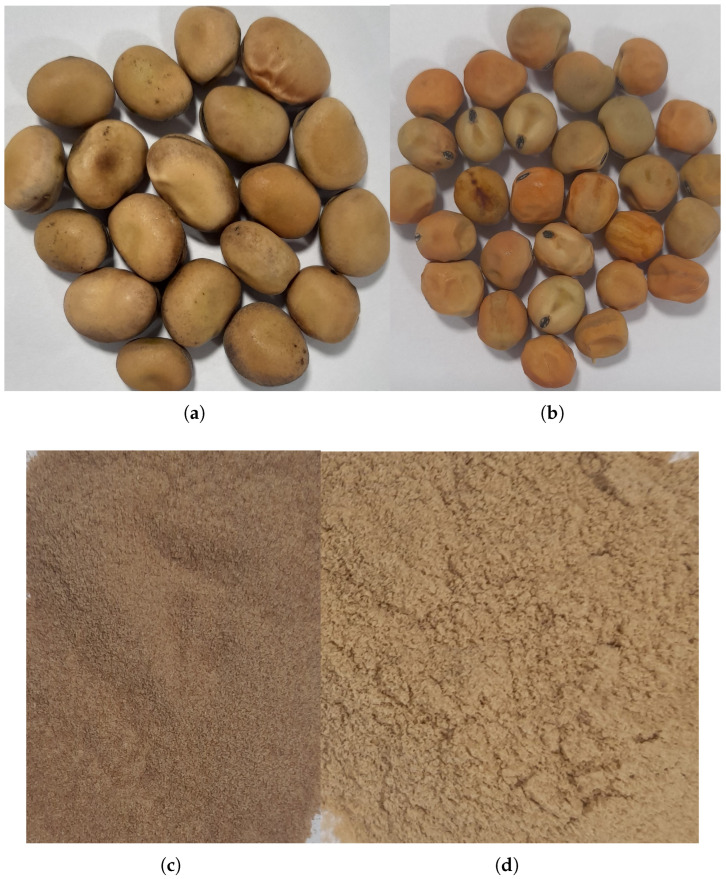
Field bean and Field pea seed and protein isolate. (**a**) dried seeds of field bean, (**b**) dried seeds of field pea, (**c**) protein isolate of field bean, (**d**) protein isolate of field pea.

**Figure 2:**
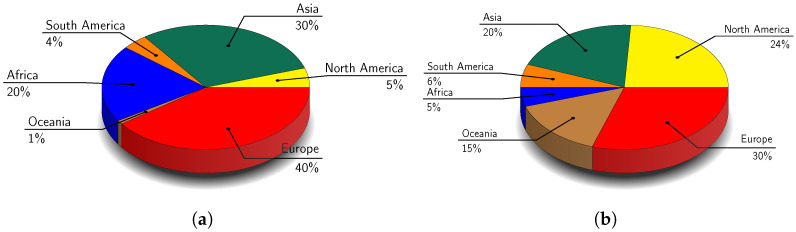
Global production distribution of field bean and field pea by region [38]. (**a**) Field bean, (**b**) Field pea.

Field bean *Vicia faba* var. *minor* (Figure 1a) ranks as the seventh most important protein source amongst grain legumes globally [28,34]. Primarily used as animal feed, it outperforms peas in protein content and resistance to certain pathogens [17]. Beyond being a rich protein source, it also contains beneficial fibres, unsaturated fatty acids, vitamins, and minerals [35]. Its improved protein content and reduced antinutritional factors, such as vicine and convicine glycosides, making it increasingly valuable for food and feed production [30]. Field beans also provide health benefits due to its bioactive compounds, which have hypocholesterolemic, immunostimulatory, and anticancer effects [36]. Moreover, field beans improve soil health through nitrogen fixation by symbiotic bacteria - rhizobia, reduced dependency on nitrogen fertilisers, and lower greenhouse gas emissions [17]. Additionally, they provide functional, nutritional, health, and environmental advantages, making them a valuable crop for sustainable agriculture [28,37]. In terms of production of field bean, Europe led the globe in output, accounting for 40% of global production of field bean (Figure 2a), followed by Asia (30%) and Africa (20%) [38].

Peas serve dual purposes as a dietary staple and as animal feed [39,40]. For human consumption, *Pisum sativum* var. *sativum* is widely used, with seeds consumed fresh, canned, or dried [39]. In contrast, the field pea (Pisum sativum var. arvense) is primarily grown for animal feed, supplying both flour and biomass [39].

The field pea (*Pisum sativum* var. *arvense*) (Figure 1b), is a cool-season legume widely cultivated for both human and animal nutrition [40,41,42]. Its seeds are rich in protein, complex carbohydrates, and micronutrients such as vitamins (e.g., B group vitamins, vitamin A and folic acid) and minerals (e.g., calcium, iron, and zinc) but low in fats with varying proximate composition across different cultivars [19,40,41,43,44]. Field peas have higher levels of lysine and tryptophan compared to cereals, and they contain fewer trypsin inhibitors (5–20%) than soy, making them nutritionally valuable [19]. Field peas are also rich in bioactive compounds like polyphenolics, especially flavonoids, which give them potential as functional foods or health products [40,43]. Additionally, field peas are beneficial for crop rotations due to their ability to fix nitrogen in the soil [10,40]. In terms of production, Europe led the world in output, accounting for 30% of global production of field pea (Figure 2b), followed by North America (24%) and Asia (20%) [38].

An increasing interest is now emerging for the utilisation of the faba beans and field peas in the food and feed industry [45]. The need for ingredients that meet vegetarian and vegan dietary requirements, particularly more protein from environmentally sustainable sources, is being driven by the growing global market for relatively new types of plant proteins. Field beans and field peas are especially suitable as a new source of protein for food applications because of their worldwide availability, nutritional value, health benefits, hypoallergenic properties, non-GMO status, and ecological advantages [11,31,32,40]. Therefore, there is significant interest in revalorizing this untapped source of sustainable dietary protein for the production of a functional protein for the food industry [2,21,46]. Additionally, field beans and field peas are an under-researched alternative pulses with great potential as functional food ingredients [35,45].

Nevertheless, field beans and field peas protein require more research to optimize protein modification methods and as a functional ingredient in various food applications to increase industrial viability. The rationale for this review is that although there are many plant proteins, the focus will be on field beans and field peas due to their high initial protein content, which makes them suitable for protein derivatization, and their functional properties, which show promise in various of applications, particularly after modification. Research on field beans and field peas is not as common than that of other types of plant proteins, such as soy, but their combination of functionality, economically good protein content, and ease of production makes them an excellent choice for industrial application. Hence, this review aims to fill this gap by providing current insights into its overall chemical composition, bioactive compounds, protein extraction process optimization, techno-functionality, and potential for protein modification of field beans and field peas protein. Furthermore, the limitations for human consumption as well as application of field beans and field peas protein in foods and health benefits are discussed.

## 2. Chemical Composition of Field Bean and Field Pea

Pulse crops, including beans, lentils, and other legumes, are highly nutritious, offering a rich source of macronutrients like protein, resistant starch, and dietary fibre, as well as micronutrients such as iron, zinc, folate, and riboflavin [43,47,48]. They also contain bioactive compounds, including polyphenols and phenolic acid, which contribute to various health benefits such as reduced risks of colorectal cancer, improved gut health, lower blood cholesterol, and reduced cardiovascular disease risk [11,43,47].

These crops provide essential amino acids such as lysine, leucine, isoleucine, and phenylalanine and are particularly effective when combined with cereals for a well-balanced protein intake [9,19]. Pulse flours, enriched with vitamins, minerals, and phytochemicals, are increasingly being utilised in food innovation to improve nutritional content and address micronutrient deficiencies [9,24].

Although pulses are nutritionally reach and inexpensive, they also contain some antinutritional factors that may require processing or special preparation techniques to mitigate [9,11,47]. Nonetheless, their contribution to both human health and sustainable food systems underscores their value in diets worldwide.

The review of the approximate composition of the field bean and field pea is shown in Table 1. Field beans are nutritionally valuable legumes, rich in proteins (22.7–35.3%), carbohydrates (41.0–51.9%), dietary fibre (7.5–16.59%), unsaturated fatty acids, vitamins (notably B-complex), and minerals [24,30,36,47]. The mineral and vitamin content of the filed bean and field pea is presented in Table 2. They also contain bioactive compounds such as flavonoids, tannins, lignins, gallic acid, stillbenes, phenolic acids, and L-DOPA (L-3,4-dihydroxy phenylalanine), which offer numerous health benefits, including antioxidant, neuroprotective, anticancer (breast, renal, and colon), and anti-inflammatory effects [6,10,24,27,29]. These properties make them suitable for the development of functional foods and nutraceuticals, especially for the management of chronic diseases such as diabetes, cardiovascular disease, and even Parkinson’s disease due to their L-DOPA content [2,29,30,36]. Despite their high yield potential and growing importance as a sustainable protein source, their cultivation remains limited to specific regions (e.g., the Middle East and Europe) due to the presence of antinutritional factors (ANF) [30]. ANFs in field beans, including lectins (0.8–3.2 HU/mg), saponins (0.02–0.12 mg/g), phytic acids (1.12–12.81 mg/g), and condensed tannins (0.27–5.89 mg/g), are listed in Table 3. These substances can decrease digestibility and have negative health effects on vulnerable people, including favism and flatulence [29]. Compared to other legumes, faba beans have a higher concentration of lectins (haemagglutinins). Similarly, faba beans contain oligosaccharides (stachyose, raffinose, and verbascose) that can ferment and cause flatulence, which can cause pain in the abdomen [29]. Processing techniques like dehulling, soaking, fermenting, or cooking can mitigate these effects, enhancing their suitability for broader consumption [29]. In general, faba beans present a promising solution to meet the growing demand for sustainable, nutrient-rich food products while addressing challenges posed by antinutritional factors.

The field pea is a significant forage crop and one of the oldest autogamous winter-season legumes. Field Peas are rich sources of nutrients, including protein (16.14–25%), carbohydrates (17.0–64.08%) in the form of starch), fat (1.5–3.0%), dietary fibre (10–20%), sugar (4–10%), vitamins, minerals (2–4%), and antioxidant components like polyphenols, ascorbic acid, phytoprostanes, and phytofurans [52,57]. Fibre content includes both insoluble (10–15%) and soluble fibre (2–9%), contributing to digestive health [40]. It has a high concentration of tryptophan and lysine amino acids and a low level of cysteine and methionine amino acids, making it a nutritious source of protein [19,53,58]. Peas also contain B group vitamins, minerals, polyphenols and non-starch carbohydrates like sucrose, oligosaccharides, and cellulose [10,40,54]. The chemical composition of field pea seeds is summarised as shown in Table 2. The most prominent mineral element present in peas is potassium (1.04%) contained in the dry and dehulled weight of the peas, followed by phosphorous (0.39%), magnesium (0.10%) and calcium (0.08%), respectively [54]. However, like other legumes the presence of antinutritional factors particularly such as tannins, proteases inhibitors, trypsin inhibitors, amylase inhibitors, lectins, polyphenolic substances, raffinose family oligosaccharides (raffinose, stachyose, and verbascose), phytic acid, haemagglutinins, glucosinolates and lectins can limit wider use of field pea seeds in both human and animal nutrition [55,59]. Antinutrients reduce the bioavailability of proteins and trace elements, that are responsible for the low protein digestibility of field pea seeds [60,61]. Variations in nutrient profiles have been linked to different cultivars, locations, several environmental/growth conditions like climate (temperature and precipitation) and soil (temperature, moisture, soil pH, and aeration), harvesting and processing methods [53,62,63]. In temperate climates, both peas and fava beans are a sustainable source of protein. Both are low in fat and offer high nutritional value, making them versatile ingredients in food and feed formulations [46]. In addition, they are known for being rich in fibre, vitamins, and substances that lower blood triglycerides and cholesterol, further contributing to cardiovascular health and the prevention of chronic diseases [16,19,30,53].

**Table 3:** Antinutrient composition (mg/g) of *Vicia faba* var. *minor* and *Pisum sativum* var. *arvense* seed reported by various authors ^a^.

	Field Bean						Field Pea	
	References							
Antinutritional Factors	[35]	[24]	[34]	[17]	[64]	[50]	[55]	[65]
Total polyphenols	10.9–19.86	1.4–5.0	2.66–2.81	NR	6.6–7.9	NR	NR	NR
Total flavonoids	5.25–6.96	NR	NR	NR	NR	NR	NR	NR
Condensed tannin	0.27–0.67	NR	1.12–1.24	NR	4.54–5.89	NR	5.24	0.34
Phytate	NR	1.12–12.81	NR	NR	NR	NR	NR	2.1
Saponins	NR	0.02–0.12	NR	0.02–0.04	NR	NR	NR	NR
Vicine	NR	0.40–7.01	NR	0.86–5.46	NR	NR	NR	NR
Convicine	NR	0.04–3.12	NR	0.52–4.02	NR	NR	NR	NR
Raffinose	NR	1.1–3.9	NR	NR	NR	1.7	37.5	10.1
Stachyose	NR	4.4–13.7	NR	NR	NR	8.1	NR	39.4
Verbascose	NR	8.0–15.0	NR	NR	NR	20	NR	39
Trypsin inhibitor (TIU/mg)	NR	1.2–23.1	NR	NR	NR	NR	1.1	0.4
Lectin (HU/mg)	NR	0.8–3.2	NR	NR	NR	NR	NR	NR

^a^ Results are expressed on a dry weight basis. NR—Not Reported.

## 3. Extraction Process of Protein

With an increasing global demand for plant-based proteins, extracting high-quality proteins from pulses has become a research and industrial priority [10]. In addition, to increasing the protein content and reducing the impact of antinutrients, proteins are extracted to produce isolates and concentrates [66]. Protein extraction aims to isolate the protein fraction while preserving valuable coproducts such as dietary fibre, vitamins, and bioactive compounds, enhancing the overall nutritional and functional value of pulse-based foods [5,67,68]. There are two types of extraction techniques: conventional extraction methods and innovative extraction methods. A long extraction time with limited extraction selectivity, poor extraction yields, high-purity solvent cost, changes in protein techno-functional properties, low stability, an undesirable colour, high solvent evaporation, and thermolabile protein degradation are the limitations of conventional approaches [69,70]. Common extraction methods include pretreatment, solubilization, and precipitation. Solubilization typically uses aqueous or alkaline conditions to maximise yield, followed by precipitation techniques such as isoelectric precipitation or ultrafiltration to concentrate and purify proteins [71]. Eco-innovative techniques are desperately needed to maximise protein extraction output without sacrificing environmental sustainability or the functional, nutritional, and technological properties of proteins [72]. Recent advances, such as enzyme-assisted extraction and membrane filtration, have improved efficiency, protein quality, and sustainability by valorizing coproducts, including fibres and starches [73,74]. The extraction of protein isolates and concentrates employs various techniques to optimise yield and maintain structural and functional properties of the protein. Among the most widely used legume-derived protein extraction methods used are dry fractionation, wet fractionation, alkaline extraction followed by isoelectric precipitation, salt extraction, dialysis, micellar precipitation, and aqueous extraction at pH levels greater than 7. Each method has distinct advantages and limitations, influencing the quality and characteristics of the resulting protein products. For example, heat treatment or enzymatic hydrolysis may destroy essential functional properties such as solubility, emulsifying ability, and gelling properties [71,72,75,76]. Since proteins impart superior functional characteristics, their extraction and purification lead to changes in their nutritional (amino acid composition), physicochemical (surface charge, surface hydrophobicity) and functional properties (water absorption capacity, oil holding capacity and solubility). According to Badjona et al. [77], these changes ultimately impact final products when they are incorporated into foods [2,24]. The choice of the extraction method significantly impacts the proteins composition, structure, and ultimately its functional properties, such as solubility, emulsification, and gelation. These properties are crucial for the applicability of protein in various food products, including meat alternatives, dairy substitutes, and protein-enriched formulations [14,75]. Furthermore, the extraction process can influence the presence of antinutritional factors and bioactive compounds, thereby affecting the nutritional profile of the extracted proteins. Techniques such as salt extraction and mild fractionation aim to mitigate these issues while maximising protein yield [10]. Understanding these extraction techniques allows food scientists and manufacturers to tailor pea protein products to meet specific nutritional and functional requirements in the ever-evolving food industry [24].

### 3.1. Preprocessing: For a Better Functionality

The main goal of pretreatments is to increase protein separation efficiency during the milling and air classification processes [78]. The preprocessing of pulse seeds includes cleaning, drying, sorting, dehulling, splitting, soaking, cooking, fermentation, and germination before protein extraction [10,44,63,79]. Pre-processing procedures can greatly improve the extraction processes effectiveness and quality. The majority of pretreatments are applied in the separation of the hulls and cotyledons, which facilitates the extraction of proteins while maintaining their techno-functional qualities [10,44]. Dehulling seeds is an optional pretreatment when flour is prepared from legume seeds [22]. This process improves colour, removes bitter or astringent components, reduces antinutritional factors such as phytic acid and condensed tannins, and slightly raises protein content [10,22,80]. By removing the fibrous seed coat digestibility, palatability, textural and cooking qualities of legume flours are potentially improved [22]. For example, dehulling is generally applied to reduce antinutritional factors present in grain legumes. However, these pretreatments can also affect the colour of the produced ingredients and process performance [80]. Dehulling, where the cotyledons are physically separated from the hulls via (air) sieving, can remove polyphenols and condensed tannins since these are mostly present in the hull of the seed. Polyphenols and condensed tannins are both ANFs and contribute to “beany” flavour perception [80]. Furthermore, pretreatment techniques like soaking, boiling, or fermentation might improve the solubility and digestibility of proteins by reducing antinutritional components found in legumes, such as oligosaccharides, tannins, and phytates [78,81,82]. For instance, chickpea seeds are soaked overnight, there is a considerable decrease in tannin concentration by 53%, along with a reduction in carbohydrate content by 20–21% [83]. On the hand, soaking chickpeas seeds for (8 h, 35 °C), exhibited increases in protein content by 1.59%, crude oil content by 18.81%, and protein digestibility by 12.81%. This treatment on chickpeas also reduced antinutritional factors such as tannin and phytate by 21.68% and 22.72%. The ash and carbohydrate contents in the chickpea were also reduced by 10.29 and 1.49% [83]. Also, soaking and heat treatments can be effective at reducing ANFs and off-flavour concentrations in pulse ingredients [80]. Pulse seeds (peas, lentils, chickpeas, fava beans and common beans) that are soaked in water (1:5 *w*/*v* seeds to water) for four hours and subsequently rinsed with the same water ratio show significant decreases in lectins, and total oxalates, but no effect on phytic acid [80]. For longer soaking times, greater phytic acid reductions have been reported: after 12 h soaking (1:5 *w*/*v* seeds to water), dehulled faba and kidney beans had 32.7% and 5.66% less phytic acid, respectively [80]. Whole chickpeas soaked for 2, 8, 12 h (1:5 *w*/*v* seeds to water) had their phytic acid content decreased by 47.4%, 49.1%, and 55.7% respectively. Longer soaking times reduce phytic acid more extensively due to the activation of endogenous plant phytases [80]. A study shows that when chickpea protein underwent milling treatment, the protein content increased by 9.3%, but the overall protein yield decreased by 48.93%. Furthermore, functional attributes such as solubility, foaming capacity, and oil holding capacity increased by 1.78%, 5.12%, and 37.33%, respectively. On the other hand, water holding capacity and emulsion activity decreased by 43.39% and 1.30% [83]. Studies indicate that the protein solubility of chickpea protein increases by 6.67% and 2.79% after 24 h of germination, compared to non-germinated seeds [83]. Another interesting preprocessing method is germination, which increases antioxidant capacity and mitigates antinutritional components while simultaneously improving the nutritional content of seed storage proteins. This is accomplished during the germination phase by activating hydrolytic enzymes [14]. The water, oil holding, and foaming capacity of germinated chickpea verities increased by 21.24%, 16.39%, and 25% in Arerti and 13.88%, 22.16%, and 39.5% in Natoli as compared to native chickpea protein [83]. On the other hand cooking the pre-soaked pulses for 1 h at 95 °C decreased the concentration of all measured ANFs. Lectin was almost entirely deactivated, and phytic acid reduction varied (10–80%) [80]. The quality and functionality of grain legumes are influenced by multiple factors, including the cultivar, agronomic conditions, maturity at harvest, and post-harvest handling practices such as storage and transport. Pre-milling treatments, milling conditions for producing pulse flours, and processes used to manufacture fractionated ingredients all play critical roles in determining the quality and functionality of the resulting protein extracts [47]. The extraction process, pH, solubilization time, number of washes, ionic strength, solvation ratio, temperature, extraction apparatus, and filtration or purification methods are some of the factors that affect the effectiveness of extraction and the properties of the protein isolate [10]. Seeds can also be preconditioned, such as soaking in water, to improve yields and overall product quality. Additional pre-milling treatments may include roasting, conventional or microwave heating, cooking, germination, and micronization using high-intensity infrared radiation. Post-milling treatments, such as heat treatment of flours, can further enhance nutritional and functional properties while minimising off-flavour development [47].

Understanding the effects of preprocessing on protein extraction is crucial, as these steps can significantly influence the compositional, nutritional, and functional characteristics of the final protein powder [14]. Various studies have focused on improving the process efficacy and protein quality of pulse seeds by optimising process parameters or applying novel techniques. However, the impact of pre-processing steps, especially dehulling and milling, on the final protein isolate remains a critical area of research that warrants further investigation [47,63,80].

### 3.2. Dry Fractionation: Air Classification and Size Reduction

Dry fractionation methods are viewed as a more sustainable alternative for producing protein-enriched fractions from pulses [84]. The dry process can produce protein-enriched flours (<40–50% protein) or protein concentrates (60–70% protein). There are two main methods for extracting plant proteins: air classification and electrostatic separation [85]. The fractionated materials can also be used as a feedstock for wet extraction to further increase the protein content to produce a “protein isolate” [47,80]. This method primarily consists of size reduction and air classification, which allows the separation of protein-rich fractions from pulse flours based on particle size and density [41,84]. Dehulling and dry milling are preprocessing techniques that optimise protein enrichment during air classification [41]. In order to create flours with fine particles, size reduction involves milling the seeds. Different milling technologies, such as roller, hammer, stone, pin, and jet milling, are employed for this purpose [79]. Pin milling is one of these methods to reduce the size of the flour particles [10]. The flour then undergoes air classification, a process in which centrifugal and gravitational forces are used to separate the coarse starch rich fraction and the fine protein rich fraction by exploiting their differing densities and aerodynamic properties [14,36,86]. In dry milling of pulses, the protein bodies and starch granules are separated from each other and from other larger cellular structures, allowing size and density-based protein-enrichment during the subsequent air classification [44]. These techniques rely on physical properties like particle size and density to fractionate protein-rich components without the use of solvents or extensive thermal treatments [18]. Protein enrichment is usually favoured by large starch granule size since proteins are located in small protein bodies. For peas, the common size of a protein body is 2 μm and the common size of a starch granule is 15 μm [44]. For faba beans, the greatest proportion of a starch granule is in the range of 7–18 μm but some literature reported values in the ranges from 25 to 40 μm [44]. The impact of milling can influence the protein separation efficiency due to differences in the particle size distribution after milling. For instance, on pea samples protein separation efficiency is higher for the fine fractions, the protein separation efficiencies exhibited higher values for the twice milled pea samples. Regarding faba beans, the effect of milling intensity on protein separation is less pronounced and no clear conclusions can be made regarding the effect of milling intensity on protein separation efficiency of faba bean fine fraction [44]. The hardness or softness of seeds, as well as their fibre, ash, and oil contents have all been identified as factors that impact the effectiveness of protein separation. For example, a high fat content limits milling to smaller particle sizes and increases the likelihood of aggregation of flour particles, which hinders separation [41,79]. During classification, the flowability or dispersibility of the flours in the air is measured by a metric called de-agglomeration [79]. Particle-particle adhesion, high humidity and size, all affect how easily flours spread; for instance, finer flour particles would disperse more readily under low pressure. Food grade flowability aids can be used to improve air classification and dispersibility of oil-rich flours [41,79]. In dry fractionation, the inherent pH and ionic strength of the seed material can influence protein charge and interactions, thereby affecting the separation efficiency. Although dry methods do not involve adjusting these parameters directly, understanding their impact is essential. For instance, proteins exhibit minimal solubility near their isoelectric point (pI), which for faba bean and pea proteins is around pH 4.5. At this pH, proteins tend to aggregate, potentially impacting the efficiency of dry separation. Therefore, ensuring that the milling and fractionation processes occur under conditions that maintain the native pH away from the pI can help preserve protein dispersibility and separation efficiency [18]. Protein-rich faba bean flour prepared by dry fractionation was reported to exhibit superior functionality, protein solubility (85% at pH 7), foaming capacity, and gelling ability compared to isolate produced through isoeectric precipitation or acid extraction [87]. Maintaining the native ionic strength of the seed material is also important, as it influences protein-protein interactions and the overall effectiveness of the fractionation process [18]. Beyond pH and ionic strength, other parameters significantly influence dry fractionation outcomes. Moisture content of the flour should be controlled within 10–12% to prevent particle agglomeration, which can hinder efficient separation [80]. Additionally, achieving a fine particle size through pin milling, typically below 50 μm, enhances the differential behavior of protein and starch particles during air classification, thereby improving the yield and purity of the protein-enriched fraction [79]. While dry fractionation minimizes alterations to protein functionality, certain technical parameters are crucial to optimize the efficiency and reproducibility of the process [18,44,87]. A novel dry fractionation technique called electrostatic separation uses electrical forces acting on charged materials to separate particles [85]. It can be used as a post-enrichment step post air classification, a pre-enrichment phase before air classification, or as an alternative for air classification [80,84]. Electrostatic separation improves protein enrichment in dry fractionation by leveraging the triboelectric properties of materials to separate proteins from starches [10,79]. Triboelectric charge characteristics brought forth by the particle-wall and particle-particle interactions are used in this method to separate the particles. These interactions give protein a positive charge while giving fibre components a minor negative charge. As a result, the protein can be separated in an electrostatic field to obtain higher protein purity than the air classification [10,84,88]. The hybrid of air classification and electrostatic separation obtained high purity protein concentrate. Electrostatic separation is an efficient and sustainable alternative for producing [41,78] high-quality protein concentrates [41,78]. However, more research is needed to improve and optimise this fractionation method [10]. Compared to wet fractionation, dry fractionation preserves the native functionality of proteins, yielding protein concentrates with desirable functional properties such as solubility, emulsification, foaming ability and foam stability and gelation [41]. However, protein purity in dry fractionation is often lower, usually around 50–60%, compared to the higher purity achieved with wet processes, and can result in lower protein solubility [14,48]. Recent advancements focus on optimising air classification parameters, pretreatments and size reduction techniques to improve protein yield and quality [79,80,84]. Additionally, new milling technologies and enhanced air classification strategies aim to increase protein recovery and minimise energy consumption [84]. Dry processing by milling and air classification can be used to produce protein concentrates with protein content up to 70%, depending on the pulse used [9]. Dry fractionation is a sustainable, cost-effective, energy-efficient method for extracting pulse proteins that maintains the native structure (charge) of the proteins while minimising water and chemical consumption [14,41,78,84].

### 3.3. Wet Extraction: Alkali Extraction/Isoelectric Precipitation (AE-IEP)

Wet extraction, also known as wet fractionation, is one of the most widely used techniques for producing protein components [41]. Various studies have explored the use of wet extraction processes to isolate proteins from legumes such as peas, lentils, and beans [84,89]. Traditionally, wet extraction techniques have been used primarily to produce protein isolates and concentrates [66]. Precipitation or ultrafiltration are the two basic methods of wet extraction. Pulse seeds are first ground into flour during the wet protein extraction process, and then they are transformed into a suspension in an acidic or alkaline environment [90]. It typically consists of an alkaline extraction, an isoelectric precipitation, and a final drying step into a fine powder (referred to as concentrate or isolate) by freeze-drying or spray-drying techniques for ease of storage and transportation [10,41]. This method apitalises on the pH-dependent solubility of proteins, where extraction at an alkaline pH solubilises most protein fractions, and subsequent precipitation at their isoelectric point enables protein isolation [75]. In contrast, albumins remain soluble across a broader pH range, allowing for effective separation and concentration of different protein fractions [75]. Proteins are solubilised at an alkaline pH (8–11) using an alkali, such as sodium hydroxide (NaOH) or potassium hydroxide (KOH) [10,41,84,91]. At this pH, the protein fractions, particularly globulins like legumin and vicilin become more soluble because they carry a net negative charge, leading to the dissociation of protein aggregates [10,41]. After the proteins are solubilised, the solution is subjected to isoelectric precipitation. This involves lowering the pH by acid-induced precipitation to the isoelectric point of the protein, usually between pH 4 and 5, where the net charge on the proteins is neutral [41,75]. At this pH, protein solubility is minimised, leading to protein aggregation and precipitation [92]. The precipitated protein is then separated by centrifugation or filtration, washed, and neutralised to produce the final protein isolate [74]. The non-protein components, such as starch, fibres, and lipids, remain insoluble and can be separated by centrifugation or filtration [14,41,80]. After neutralisation, the precipitated protein is dried, either by spray drying or freeze drying to obtain protein concentrates or isolates with purities between 80% and 90%, respectively, on a dry weight basis. This step optimises protein extraction while preserving key functional properties such as emulsification and gelation [41]. A number of factors, including particle size, solvent type, solubilisation pH, temperature, extraction duration, and flour-to-solvent ratio, influence protein yield [10,41,75,80]. Furthermore, factors like the concentration of the alkaline solution and the length of processing have a big impact on protein recovery [41,75]. The AE-IEP process offers several advantages that have contributed to its widespread use in the food industry. These are AE-IEP can produce protein isolates with over 80–90% protein content, which makes it highly suitable for applications where high protein levels are desired [75]. Proteins isolated through AE-IEP generally exhibit good solubility, emulsification, and foaming properties, making them suitable for various food applications, including dairy and meat analogues [14,84]. Alkaline conditions and subsequent processing steps can help reduce or eliminate certain antinutritional factors, such as phytates, tannins, and protease inhibitors, improving the nutritional quality of the protein isolate [75,84]. AE-IEP has a number of drawbacks despite its benefits, such as the loss of sulfur-containing amino acids and the decreased bioavailability of important amino acids such as histidine (80%) and sulfur-containing amino acids (71%). The protein composition and quality can also be changed by the conversion of cysteine and serine residues into dehydroalanine, which can then react with lysine. Achieving a balance between low functional and nutritional losses and high protein yield requires optimising these factors [41,84]. Furthermore, a major challenge is the possibility of protein denaturation, which could impact its functionality, particularly its solubility and digestibility [41,78]. In addition, alkaline conditions can cause undesirable reactions like Maillard browning, which would change the protein isolate’s colour and flavour. The objective of promoting a sustainable food system is contradicted by the high energy, water, and chemical resources used in wet extraction [20,78,79]. The techno-functional properties of protein isolates obtained via alkali extraction and isoelectric precipitation are often suitable for applications in food products that require emulsification, foaming, and gelling capabilities. However, the loss of solubility during isoelectric precipitation can limit the use of these proteins in beverages or other liquid food systems [9,10,14]. To address this, additional processing steps such as enzymatic hydrolysis or heat treatments may be applied to enhance solubility without compromising functionality [9,93].

### 3.4. Ultrafiltration Processing

Ultrafiltration processing (UF) is a pressure-driven non-thermal membrane filtration process that is frequently used as an alternative to isoelectric precipitation following alkaline extraction. It has uses in protein fractionation, concentration, desalting, and clarification [41,80]. UF is a technique for separating dissolved proteins that uses a selectively semipermeable membrane. The protein solution is passed through ultrafiltration membranes with pores that allow smaller soluble substances, such as carbohydrates, to flow through while selectively retaining proteins [83]. Ultrafiltration preserves the natural structure and functions of the protein, making it a gentle process. Because it does not employ hazardous chemicals or effluents, it can be considered a green technique [41]. In order to preserve proteins of interest, the UF method isolates proteins according to their molecular size, which ranges from 1000 to 100,000 kDa. Different molecular weight cut-offs are available [10,80]. A molecular weight cut-off is a common characteristic of membrane UF technology, which uses membranes with opening diameters of 0.001–0.1 μm that function as physical sieves that can retain molecules with a molecular weight of about 30,000 kDa [41]. To obtain fractions with distinct sizes, the solubilised protein is sequentially passed through a smaller membrane (e.g., 10 kDa), and the permeate is collected as the <10 kDa fraction. The retained solution is further passed through a bigger membrane size (e.g., 30 kDa), and the permeate is collected as the 10–30 kDa fraction while the retentate is the >30 kDa fraction. A reversed technique could start with a larger molecular membrane and the retentate collected from one size to the other [41].

In order to provide components with a high native content and usefulness, membrane UF is utilised in combination with other protein extraction methods [41]. In order to increase product recovery and purity, diafiltration, which dilutes the retentate by adding water, is frequently used in conjunction with UF [80]. Diafiltration is a technique used to decrease the viscosity of the solution and speed up membrane penetration by periodically adding distilled water to the retentate [41,88]. Following mild wet fractionation, proteins in the supernatant can also be concentrated by ultrafiltration or diafiltration. To increase protein yield from roughly 78% to 87%, several washing stages and ultrafiltration were applied to the protein and starch-rich fractions [88]. In general, UF is a new application that produces high-purity, high-quality components for pulse protein processing. UF is also applicable to industry because it may be utilised widely. By eliminating contaminants such as fibre and carbohydrates and concentrating protein-rich streams, UF can produce high-purity protein fractions (90–95%). The study showed that UF produced the highest protein content and purity at pH values of 9 and 6. At a pH of 9, DF and UF resulted in a lower phenol concentration. But the levels of trypsin inhibitors were high in both techniques [83]. Being a non-thermal procedure, UF aids in keeping the natural structure of proteins, protecting their ability to dissolve, emulsify, and gel [41,80]. Overall, UF/diafiltration, especially when combined with alkaline extraction/isoelectric precipitation, has shown potential to enhance protein extraction yield and functionality. These techniques also inactivate protease and amylase inhibitors while reducing lectins and anti-nutritional factors [70]. Proteins from peas, chickpeas, and lentils were extracted using alkali-combined ultrafiltration in earlier research. The findings demonstrated the good water-holding capabilities, emulsifying, foaming, and gelling qualities of pea, chickpea, and lentil proteins [76]. According to Asen et al. [41], UF is a non-invasive, user friendly, environmentally friendly technology that has demonstrated efficacy in protein processing [41]. A significant challenge of UF is membrane fouling and concentration polarisation, where particles accumulate on the membrane surface, reducing efficiency and increasing operational costs. Fouling reduces the efficiency of the process and reduces protein yield, and the remedy is the selection of appropriate membranes for protein separation [41].

### 3.5. Salt Extraction and Micellization

Salt extraction and micellization is a novel method of extracting pulse proteins that separates proteins based on their solubility in salt solutions. This process involves the principles of salt-in (solubilisation) and salt-out (concentration) proteins, followed by a desalting process that reduces the ionic strength of the protein environment [10,74,83]. According to Asen et al. [41], dialysis or membrane ultrafiltration could be used in place of the concentration stage of salting out. When pulse flours are dissolved in saline solutions, proteins dissolve, while fibre, carbohydrates, and other components do not. Micellar or salt-induced extraction and precipitation use a solution with a high ionic strength to solubilise proteins while avoiding extremely high or low pH levels or elevated temperature are not required. The proteins are then precipitated and aggregated by further dilution. It is believed that the proteins are shielded from complete denaturation by residual ionic strength [10,88]. Proteins typically exhibit salting-in at low ionic strengths, 0.1–1 M. The extraction takes place at the natural pH level of 5.5–6.5. A slightly higher legumin concentration is often extracted via alkaline extraction. However, because legumin is less soluble in diluted salt solution than vicilin, salt extraction is a better method for obtaining vicilin and convicilin [10]. Protein isolates from salt extraction often showed lower water holding capacity but higher solubility, oil holding capacity, and foaming capacity than others. According to Shanthakumar et al. [10], isolates with higher extraction yield and functionality are produced using salt extraction. Furthermore, the salt extraction approach had the advantages of preserving the original structure of the protein and preventing denaturation, but also had the drawback of having a low protein extraction rate and purity [76].

A mild extraction technique called micellar precipitation yields proteins with a high concentration of native structure. The micellization method induces precipitation of protein and micelle formation, which occurs by adding cold water in the ratio of 1:3 to 1:10 (*v*/*v*) of high salt protein extract to water [10,41]. Dilution of the protein solution forces solubilised proteins to regulate the low ionic strength through a series of dissociation reactions to form lower molecular aggregates. When it reaches a critical protein concentration, the aggregates combine into a comparatively low molecular weight micelles, precipitated from solutions. Micelles are nanoscale aggregates that form in water, with the hydrophobic moieties in the centre and the polar heads oriented toward the surrounding environment. To maximise micelle formation, the diluted solution is left to stand for some time [10,41]. Centrifugation is used to separate the insoluble components after the proteins have been extracted in a neutral pH salt solution. Reverse micellar precipitation is a further variation of this technique that creates nanostructured aggregates of surfactant molecules in a non-polar environment with water in the centre of the structure [41]. One advantage of the micellization process is that it is milder and involves less drastic pH shifts, which means that less protein is denaturated during the process. Due to a lack of protein solubilisation, the micellization approach has a low protein recovery rate [10]. Another approach is to extract using only water. The extraction process is frequently repeated since the yield from a water-only technique is lower than with an adjusted (more alkaline) pH or ionic strength. Proteins were first extracted at a higher pH, then purified and concentrated using ultrafiltration in a different technique that did not include protein precipitation. They demonstrated how albumins, which often do not precipitate due to their superior water solubility and are thus lost, may be recovered from raw materials by employing ultrafiltration to bypass a precipitation phase. In terms of emulsion and foam stability, the fractions produced by such a gentle technique have essentially distinct characteristics [88].

### 3.6. Mild Fractionation

A mild fractionation process is proposed to produce protein isolates using a hybrid approach [94]. For instance, after dry fractionation, the fine fraction of pea flour was recovered and suspended in water. It was then separated layer by layer using centrifugation forces or, if necessary, further purification (such as dialysis or ultrafiltration) to increase purity (up to 75–90 g protein/100 g dry matter). Dry and mild fractionations both need physical separation according to density distribution and size. The yields of the dry method, which requires no water, depend on the number of passages (milling-air classification) while maintaining its native form [14]. Conversely, wet processing decreases the amount of non-protein materials, yields a more pure protein isolate (80–90% protein), and uses a lot of water, chemicals, and energy, but also decreases native functionality [14,88]. However, it was discovered that the modestly fractionated protein concentrates performed better overall than the heavily fractionated ones. Furthermore, it is possible to optimise the protein composition for particular functional behaviour by utilising fractionation procedures. Previous research showed that fractionation could impact the functional characteristics of pea protein because they found that the extent of fractionation could change the protein composition and viscosity of the fractions and that the limited fractionated samples had a better gelling capacity [94]. These three protein fractions an albumin-enriched fraction, a globulin-enriched fraction, and a fraction containing both globulins and albumins, could be produced using a standard aqueous fractionation procedure. The ability of globulins to precipitate at pH 4.5 is the basis for the separation of these proteins [94].

### 3.7. Ultrasound-Assisted Extraction

Ultrasound-assisted extraction (UAE) is an efficient, eco-innovative and promising method for enhancing protein yield and functionality from peas and beans. UAE uses cavitation-induced shear forces to break down plant cell walls, enhancing solvent penetration and protein release. This technique significantly reduces extraction time, temperature, cost, and environmental impact compared to traditional methods [23,95,96]. UAE works by generating high-intensity sound waves that collapse microbubbles, leading to cell wall disruption and improved protein extractability [95,96]. Ultrasound has also various effects on the bioactivity and functionality of food proteins [97]. Various studies on protein isolation have been conducted using different sources, with ultrasound serving as an effective technique. The most common ultrasound frequencies used in food processing range between 20 and 100 kHz and are known as high-power ultrasound waves [98,99]. Ultrasound is an effective tool for protein extraction only when suitable frequencies are applied. Hence, the selection and application of appropriate ultrasound frequencies are the most critical factors in protein extraction. Numerous studies have also shown that using ultrasound as a pretreatment increased protein yield or protein release rate [98,99]. In the case of faba beans, optimized UAE conditions led to a protein extraction yield of 19.75%, surpassing the 16.41% yield obtained through conventional methods. The extracted proteins exhibited superior water and oil holding capacities, with minimal impact on thermal properties [95]. Additionally, field bean protein isolates obtained via UAE showed a 76.84% improvement in extraction yield, with enhanced functional properties suitable for food applications [23]. Similarly, research on chickpea proteins demonstrated that UAE achieved a higher extraction yield (66.1%) compared to conventional alkaline methods (55.1%), while also enhancing water and oil absorption capacities, as well as foaming properties [100]. Additionally, UAE reduces antinutritional factors (e.g., phytates) in beans, enhancing protein bioavailability [23]. Several ultrasound- and sample-related process factors affect the efficacy of the ultrasound [100]. The energy density, ultrasound intensity and treatment time are the ultrasound-related factors, while the type of sample/protein and sample/solvent ratio are sample-related parameters [100]. UAE poses challenges for various protein sources and process parameters. However, optimizing the process for ultrasound-assisted extraction remains limited [95]. Therefore, optimization based on the different plant sources is needed [100]. Effective UAE can lead to improved extraction yields and modified functional properties of proteins. Successful protein extraction relies on understanding how UAE parameters such as time, power, frequency, solvent-to-sample ratio, and temperature affect the extraction of faba bean proteins [68,95,98]. Effective UAE applications rely on proper optimization of these parameters, which can significantly impact both the protein extraction yield and the functional characteristics of the resulting protein concentrate [68,99,101]. High-intensity ultrasound waves can be utilized for emulsification, diffusion, and extraction, whereas low-intensity ultrasound waves are typically used in nondestructive procedures [68]. Ultrasound treatment enhances the emulsifying properties of proteins by creating turbulence that improves protein adsorption at the oil-water interface. It also increases solubility and hydrophobicity while promoting protein denaturation, exposing hydrophobic groups, and reducing water-holding capacity. This process improves oil-holding capacity through physical entrapment. Additionally, ultrasound enhances foaming capacity and stability by promoting homogenization, increasing solubility, and reducing particle size [99]. The disadvantages of UAE include the denaturation of soluble protein fractions, which reduces the protein concentration, and its high energy consumption, which is thought to be a barrier to its industrial application In addition, high energy consumption is considered a limitation for the use of UAE on an industrial scale [68,98,100].

### 3.8. Enzyme-Assisted Extraction (EAE)

Protein extraction from various plant sources is aided by the ecologically friendly green processing method known as enzyme-assisted extraction. By hydrolyzing the plant cell wall and the proteins therein, a variety of food grade protease and carbohydrase preparations, including pepsin and pancreatic enzymes, have been used to facilitate protein extraction and solubilisation from various plant sources [77,83,102]. Stiff cell walls make it difficult to remove cellular proteins. Hemicellulose, cellulose, and pectin are the components of cell walls that EAE primarily aims to break down enzymatically in order to damage the integrity of the walls [70]. The specialized functions of pectinases and carbohydrases in the cell wall facilitate the release of cellular proteins from legume seeds [68]. According to Badjona et al. [77], Rashwan et al. [72], this results in an increased protein yield and better functional qualities, including solubility. The optimal pH is maintained by proteases to avoid denaturing proteins. A typical protease enzyme/substrate concentration of 1% to 5% is optimal for a range of extraction methods. These enzymes can also prevent interactions between the released proteins and various cellular components, including phytates and carbohydrates, under specific physiological conditions. For enzymes to function at their best, a variety of acidic and alkaline environments are necessary. Although some proteases prefer somewhat alkaline environments, carbohydrases often function best in mildly acidic environments. For proteases functioning in an alkaline environment, the optimal pH and temperature ranges are 8–10 and 45–60 °C, respectively [68,98]. In contrast to chemical and physical extraction methods, EAE’s mild operating conditions, low energy consumption, and decreased waste formation led researchers to use it [68,77,102]. When protease-assisted extraction is employed, EAE can improve the nutritional (digestibility) and technological and biofunctional properties of the extracted proteins in addition to aiding in protein recovery. Plant cell wall disruption can be improved by combining EAE with physical methods, such as ultrasonic processing, for biomass pre-treatment. This will increase the efficiency of protein extraction/high protein extraction rate. This method can help make the extraction process more economically feasible by using less energy and enzyme, which lowers the extraction process’s total cost [76,102,103]. Despite its advantage, EAE requires specific enzymes, which can be expensive and have a long reaction time, especially at industrial scales [76]. Moreover, enzymatic activity needs to be tightly controlled to avoid incomplete protein extraction or unwanted degradation [102]. The maintenance of optimal conditions such as pH, temperature, and enzyme concentration adds complexity to the extraction process. Deviation from these conditions can lead to inconsistent results and affect protein functionality [68].

### 3.9. Fermentation

An innovative method for extracting and modifying pulse proteins is fermentation. This technique uses microbial action, usually in the form of bacteria, yeast, or fungi, to break down antinutritional components, modify the structure of the protein, and improve the digestibility and techno-functional properties of pulse proteins [3,104]. In addition to extracting proteins, fermentation improves their nutritional profile by reducing substances such as phytic acid and increasing the bioaccessibility and bioavailability of essential amino acids [104]. Legumes protein composition and structure can be affected by lactic acid fermentation. This is explained by the mechanism of the bacteria’s proteolytic activity during fermentation, which breaks down the polypeptide chain and forms new, lower molecular weight polypeptides. Protein structure and conformational variations impact the end products nutritional value and functionality [104]. Many studies have highlighted the benefits of LAB species, including *Streptococcus thermophilus*, *Lactobacillus delbrueckii* subsp. *bulgaricus*, *Lactobacillus acidophilus*, *Lactobacillus helveticus*, and *Lactobacillus plantarum*, on the organoleptic characteristics of legume protein [104]. The growth of LAB during the fermentation of pulse proteins contributes to the enhancement of flavour and aroma by hiding undesirable green notes or decreasing the presence of compounds that cause off-flavours [14,104]. Given the benefits of LAB fermentation for legume characteristics and the pH drop caused by lactic acid production, an alternate protein extraction technique based on alkaline solubilization/isoelectric precipitation was used, in which lactic fermentation rather than the addition of mineral acid produced the pH drop [104]. The advantages, limitations, functional characteristics, applications and future research trends of pulse protein extraction methods are summarized in Table 4.

## 4. Protein Fractions

The protein subunit is of crucial importance since its investigation can disclose the composition and corresponding functionality of seed storage proteins. Furthermore, this supports research on the nutritional value of protein in human diets and animal feed, as well as breeding goals for enhancing the protein composition of pulses [57,77]. The Osborne fractions of pulse proteins are as follows: (i) salt-soluble globulin fraction; (ii) water-soluble albumin fraction; (iii) prolamins, which dissolve in a solution of ethanol and water; and (iv) insoluble glutelins [48,53]. The dominant protein fractions in pulses are globulins and albumins [53,77]. Typically, globulins are present in higher amounts than albumins, affecting the rheological and textural properties of the proteins [31,105]. However, the relative amounts can vary considerably between different pulses, and also due to variety and cultivation conditions, and the albumin/globulin ratio has been reported as high as 0.5 [9]. Protein fraction and their composition, molecular structure, charge distribution determine protein isolate physical and chemical properties [53]. In globulins, there are legumin and vicilin (salt soluble proteins) comprising of high molecular weight and complex structures but their ratio plays a vital role in utilizing these proteins for fortification in various products like breads and biscuits [29,106]. The storage proteins contain two subunits one is globulins, having 7–11 Svedberg unit (S) fractions but lacks sulphur containing amino acids, and the other one is prolamins, having trypsin inhibitors and phytolectins, comprising of sulphur containing amino acids, with minor amounts of convicilin [106,107]. However, the faba bean is classified into two type’s likely globulins and non-globulins [105].

### 4.1. Globulins

In peas, the globulin-to-albumin ratio ranges from 55 to 80%, while in faba bean seeds, globulins make up 70 to 80% of the storage protein [75,77]. Globulins, a major class of pulse proteins, are categorised by sedimentation coefficient into legumin (11 S), vicilin (7 S), and convicilin [63]. Legumin and vicilin dominate faba bean and pea proteins, typically in a 2:1 ratio, with legumin being richer in sulfur-containing amino acids [2,7,16,46,48,53,77,108]. Legumin is a hexamer (∼340–400 kDa (Kilo Dalton)) composed of six subunits (60–65 kDa), has an acidic (∼40 kDa) and a basic (∼20 kDa) linked via disulfide bonds, with hydrophilic and hydrophobic regions affecting its water interaction [9,53]. In fava bean, globulins (legumin, vicilin, convicilin) are predominant seed storage proteins (approximately 85% *w*/*w*) which exist in different structural conformations, with legumin constituting about 50% of the storage proteins [77,109]. The legumin-to-vicilin ratio varies among different genotypes, typically ranging from 1:1 to 3:1 [46]. Legumin exists in two subtypes, A (∼38–40 kDa) methionine-rich and B (∼23 kDa) methionine-lacking, and is encoded by multiple gene families, each containing α- and β-chains linked by disulfide bridges [46,77,105]. These subunits exhibit heterogeneity, with molecular weights around 75 and 80 kDa [46,105]. Vicilin is a trimeric protein (∼175–180 kDa) composed of heterogeneous polypeptides without cysteine residues, preventing the formation of disulfide bonds. Its subunits (∼50 kDa) are glycosylated, hydrophilic, and cleavable into low-molecular-weight fragments. Vicilin shares structural similarities with convicilin [53]. Convicilin (8 S, 180–210 kDa), a polymorphic form of vicilin, shares 80% amino acid sequence homology with uncleaved vicilin but differs by its highly charged N-terminal extension and the presence of a single cysteine residue, enabling potential disulfide linkages. Both proteins are stabilised by noncovalent interactions, with structural differences influencing their properties. Convicilin’s unique features, like its cysteine content, set it apart functionally and structurally from vicilin [53,110]. Legumin and vicilin proteins exhibit varying solubility, emulsifying properties, and structural responses to pH and ionic strength [53]. Their functional and nutritional properties depend on their composition, structural differences, and the legumin/vicilin ratio, which processing methods like isoelectric precipitation can alter. These structural and surface properties are critical for understanding pulse protein functionality [9]. The thermal properties of faba bean proteins show that 7 S proteins denature at 84 °C, while 11 S proteins denature at 95 °C. Faba bean proteins, particularly legumin, have a high degree of structural homology with vicilin and are part of the cupin superfamily [27]. Despite their abundance, faba bean proteins are less soluble and functional compared to animal proteins, limiting their widespread use in food products. Isoelectric precipitation can be used to isolate these proteins based on their isoelectric points. The isoelectric point of convicilin is 5.5, while that of vicilin is 4.8 [77]. In field peas, the globulin fraction is mainly composed of legumin (11 S), vicilin (7 S) and convicilin (7 S), with a typical legumin-to-vicilin ratio close to 2:1 [104,111]. Pea legumin is hexamer with a molecular weight (Mw) of ∼300–400 kDa. Legumin has a 40 kDa acidic–20 kDa basic, (α-β) subunits which is linked by a covalent disulfide bond representing one monomer within a quaternary structure linked with non-covalent bond [106]. Vicilin is a trimeric protein of 170 kDa that lacks cysteine residues and hence cannot form disulfide bonds. Each monomer (∼50 kDa) can undergo post-translational cleavage, resulting in polypeptides of approximately 20 kDa (α), 13 kDa (β), and 12–16 kDa (γ). Additionally, the γ-subunit near the C terminus is sometimes N-glycosylated, while no glycosylation has been observed in legumin [104,111]. Vicilin consists of low amounts of sulfur-containing amino acids such as tryptophan, methionine, and cysteine, and higher amounts of basic and acidic amino acids such as arginine, lysine, aspartic acid, and glutamic acid amino acids. N-terminal amino groups are typically represented by aspartic acid, glutamic acid, and serine [104,111]. Convicilin has a subunit of ∼71 kDa and a molecular weight in its native form of 290 kDa, which can form trimers of three convicilin molecules or heteromeric trimers with vicilin [104,106]. Convicilin contains sulfur-containing amino acids and a highly charged N-terminal extension, thus the amino acid profile of convicilin is different than both vicilin and legumin. Within this group of proteins, two major fractions were identified: a larger albumin protein comprised of two polypeptides with a molecular mass of ∼25 kDa and a minor fraction with a molecular mass of ∼6 kDa [106]. The legumin fraction has the higher emulsifying and foaming stability compared to vicilin. Moreover, the nutritional values of legumin and vicilin are different. Vicilin has higher amounts in arginine, isoleucine, leucine, phenylalanine, and lysine compared to legumin, while the latter is richer in sulfur-containing amino acids. Thus, the 11 S/7 S ratio may play an important role in the functionality and nutritive value of pea proteins, which are critical for their performance in food formulations [112]. The concentration and types of globulins vary across legume species and varieties, influencing their nutritional value and functional properties [77]. It is also important to note that faba bean protein are not a complete protein due to their deficiency in some essential amino acids, specifically methionine and tryptophan [63]. Understanding globulins composition and functionality is crucial for enhancing legume crops and improving their applications in food and animal feed [77]. Protein structure can vary significantly depending on the extraction method used, as well as extrinsic factors such as temperature, pH, salt concentration, and ingredient modifications, all of which alter the protein’s surface characteristics and conformational stability, ultimately influencing functionality [106]. Additionally, variations in the ratios of protein fractions like legumin and vicilin are determined by genetic factors, environmental conditions, and processing techniques, leading to differences in functional performance [21,80]. For instance, a higher legumin content is generally linked to improved gelation, whereas elevated vicilin levels enhance emulsifying properties. As such, understanding and strategically modifying the globulin-to-albumin ratios in field peas and faba beans is essential for designing plant-based protein ingredients with targeted functional properties for diverse food applications. A higher globulin/albumin ratio is often associated with better protein stability and gelling properties, making legumes suitable for food processing. However, excessive globulin content may reduce protein solubility and digestibility due to its compact structure. Recent studies suggest that field peas have a more balanced globulin/albumin ratio compared to field beans, contributing to their higher protein bioavailability [53]. Understanding globulins nutritional, functional, and technological properties is crucial for enhancing legume crops and improving their applications in food and animal feed [77].

### 4.2. Non-Globulin Proteins

The majority of faba bean seed albumins are metabolic proteins that have the ability to function as enzymes. These proteins include Bowman-Brik inhibitors, albumin-2, defensins, protease inhibitors, and lectins. In comparison to other seed proteins, the albumin fraction contains significant levels of sulfur-containing acid [77]. Albumins in faba beans comprising approximately 10–20% of storage protein [46]. Pea albumins (2 S), which are water-soluble and represent 18 to 25% of total pea seed protein, are primarily metabolic and enzymatic proteins with roles in seed germination [10,53]. They include proteins such as PA-2, PA-1, lipoxygenase, protease inhibitors, phytate, α-galactosides and lectins [9,53,75]. Two small molecular weight albumins, PA1a (∼6 kDa, has 53 amino acids) and PA1b (∼4 kDa, has 37 amino acids), have been characterised [9]. The molecular weight of albumins PA1a and PA1b have exceptionally high levels of cysteine (7.5% and 16.2%, respectively), and PA1b has the ability to act as an insecticide in biological control [10]. Despite their functional and nutritional potential, albumins are often discarded due to their lower protein content and the presence of anti-nutritional compounds [75]. Albumins contain higher concentrations of the essential amino acids such as lysine, methionine tryptophan, and threonine [104,106]. Overall, albumins are vital for the nutritional and functional characteristics of legumes and play a supporting role in various biological processes in seeds [77]. Prolamin is another group of plant storage protein and presents a small amount in faba bean and pea seeds. Prolamins are alcohol-soluble and characterised by a high content of proline, glutamic acid, and leucine, while lacking lysine and tryptophan. They dissolve in ethanol/water (70–80%) and propan-1-ol/water mixtures, light acid, and alkaline solutions [10,53]. Prolamin does not coagulate by heat but hydrolyzes to proline and ammonia [10]. Similarly, glutelins, a prolamin-like protein class, have a similar amino acid profile but are more soluble in dilute bases (sodium hydroxide) or acids, chaotropic or reducing agents, and surfactants, and contain high levels of glycine, histidine, and methionine [10,53]. Glutelins, found primarily in seed endosperms, are minor components in peas, but are significant in protein composites like gluten [53]. Electrophoretic analyses, such as size-exclusion high-performance liquid chromatography (SDS-PAGE), have demonstrated that under non-reducing conditions, faba bean glutelin exhibits a wider range of polypeptides (∼12–104 kDa) compared to globulin (∼12–82 kDa) and albumin (∼12–41 kDa) fractions [46,113]. In general, albumins are vital for the nutritional and functional characteristics of legumes and play a supporting role in various biological processes in seeds [77].

### 4.3. Others Compounds

Anti-nutritional compounds are considered non-nutritive compounds since they interfere with nutrient availabilities or cause host digestive discomfort or health problems [75,114]. For example, low molecular weight proteins called trypsin inhibitors can bind to the digestive enzyme trypsin and render it inactive, lowering protein digestibility, amino acid absorption, and mineral availability. The second most prevalent soluble carbohydrate in legumes is the α-galactosides of sucrose, sometimes referred to as the raffinose family of oligosaccharides (RFOs). Due to their fermentation by gut bacteria in the large intestine, α-galactosides cause flatulence and digestive discomfort [75]. Conversely, phytic acid affects the solubility of proteins by binding different molecules, including proteins, through a variety of interactions. Enzymes and specific minerals can also be bound by phytic acid, which decreases the gut’s ability to absorb nutrients. For those who consume a lot of pulses each day, this can result in iron deficiency anaemia [75].

### 4.4. Protein Concentrate

The primary objective of producing protein concentrates and isolates is to improve the usability of the product by removing non-protein components and increasing the concentration of proteins. This enables the use of a smaller amount of protein in food formulation to provide particular functional and nutritional properties [63,68]. Protein concentrates, which can be derived from a variety of sources such as dairy products, plants, and insects, offer an option. According to Fatima et al. [68] the food industry uses these concentrates to formulate functional foods, enhance dietary profiles, and satisfy the growing demand for plant-based and alternative protein sources. Faba bean and pea protein ingredients, available as flours, concentrates, or isolates, are widely used in the food industry to enhance nutritional profiles and improve sensory characteristics such as texture, structure, taste and colour [9,14,19]. Legumes, such as peas and faba beans, are emerging as cost-effective, high-protein alternatives to dominant sources like soy, whey, and wheat. They are ideal for producing protein concentrates and isolates due to their high protein content, affordability, low allergenicity, non-GMO status, and nearly complete essential amino acid profile. This makes them increasingly popular in the market [19,27,94]. Pulse protein concentrate is produced through dehulling and milling, often without defatting due to its low fat content. Protein concentrates are by-products of air classification and include more protein than raw samples [68]. For instance, processing methods have achieved protein-rich faba bean flours with up to 65% protein content and eco-friendly concentrates with 56% protein, showcasing promising functional properties for food applications [77]. Furthermore, pea protein concentrate is typically produced via water-based physical separation and contains up to 85% protein, including a high proportion of branched-chain amino acids (BCAAs), making it suitable for sports nutrition, meal replacements, and infant formulas. Its functionality can be enhanced through “green” modifications; for example, enzymatic crosslinking with transglutaminase has been shown to double its water-holding capacity, while conjugation with polysaccharides like guar gum improves emulsifying properties [115]. Additionally, faba bean protein concentrate, produced via dry fractionation, offers approximately 61% protein content and is rich in fiber. It exhibits excellent solubility, making it ideal for applications such as protein-enriched smoothies and vegan dairy drinks. Dry-heat treatment of faba baen protein concentrate can enhance its water-holding capacity by inducing partial protein denaturation, which exposes hydrophobic sites and leads to protein aggregation, thereby improving its functional properties [116].

### 4.5. Protein Isolate

Proteins are solubilised in an aqueous medium by changing the pH to a basic medium (sodium hydroxide) to create protein isolates and contain more protein than concentrates [68]. Protein isolates are the refined forms of protein containing a substantial amount of protein with better digestibility. They are increasingly used in food and non-food applications [117]. Plant-based protein isolates, such as the pea protein isolate (PPI) and the faba bean protein isolate, are produced using methods such as alkaline extraction/isoelectric precipitation (AE-IEP), salt salt extraction with subsequent micellization, ultrafiltration yielding high protein content (80–94% for PPI and >90% for faba bean isolates) [77,117,118]. Faba bean isolates are derived from dehulled, defatted material to improve extraction efficiency by minimising lipid-protein interactions. Extraction methods significantly influence the functional and physicochemical properties of proteins like globulin, legumin, and vicilin, as well as the purity and techno-functionality of the isolates [77]. The functional properties and purity of the isolate produced can differ significantly depending on the extraction technique and conditions used [9,77]. To generate the desired protein isolate, it is necessary to optimise extraction conditions, including temperature, pH, solvent ratio, extraction time, centrifugation time, and drying conditions. When compared to raw faba bean, alkaline/isoelectric precipitation has been demonstrated to diminish favism caused by the aglycones vicine and convicine in protein isolates by 99% [77]. A key advantage of isolation is the reduction of antinutrients, with residual glycosides like vicine and convicine reduced to less than 1% [77]. Pulse protein isolates, including those from peas and faba beans, are valued for their techno-functional properties, such as solubility, emulsification, and foaming, making them promising alternatives to animal-based proteins. However, solubility near the isoelectric point remains a challenge, particularly in mildly acidic food and beverage applications [9].

## 5. Nutritional, Digestibility, and Amino Acid Distribution

Faba bean and field pea are a nutrient-dense legumes known for their rich protein content and versatile use in both food and feed. They are highly valued for their well-balanced amino acid composition, particularly its high levels of lysine, an essential amino acid often deficient in cereal-based diets [17,19,27]. A summary of amino acids is presented in (Table 5). They are increasingly valued in sustainable, plant-based food formulations due to their digestibility and nutritional benefits, though methionine supplementation is often necessary [40,43,77]. Field peas and field beans are also rich in bioactive compounds such as peptides, phenolic compounds, saponins, flavonoids and L-DOPA (levodopa), they provide health benefits including immunostimulatory, anticarcinogenic, anti-industrial bacterial, cholesterol-lowering properties, and hypocholesterolaemic properties [10,36,40,43]. These seeds also provide significant amounts of dietary fibre, unsaturated fatty acids, vitamins, and minerals, making them a promising source of plant-based nutrition [35,41]. The pea protein is a good source of small bioactive peptides that have the potential to have positive health effects by inhibiting the angiotensin I-converting enzyme (ACE) and providing antioxidant action. Peas’ high protein content may have an appetite-suppressing impact by delaying stomach emptying, reducing the absorption and concentration of glucose, and inducing the release of hormones that control hunger [10]. The digestibility of field bean and field pea proteins is relatively high, but the presence of antinutritional compounds such as tannins, phytic acid, and protease inhibitors, vicine and convicine, can reduce protein absorption and bioavailability. The digestibility of pulse seed proteins has been improved through processing methods such as dehulling, fermentation, enzymatic, and heat treatments, which help reduce antinutritional factors and improve digestibility. Otherwise, these factors can limit the bioavailability of amino acids and decrease digestibility [31]. Studies have shown that post-fermentation, pea proteins exhibit improved in vitro digestibility and an increase in amino acids like cysteine and methionine, which are crucial for protein quality [75,104]. Research is focused on enhancing protein quality and functionality through advanced processing methods. Improving the quality of plant and alternative proteins involves optimising their amino acid composition, reducing anti-nutritional components, and ensuring efficient gastrointestinal digestion for optimal biological function [15].

## 6. Techno Functional Properties

Pulse proteins are increasingly utilized in the food industry due to their functional properties [6]. The physicochemical and functional properties of protein are classified into three groups based on mechanism of their action: (i) hydration-related qualities (e.g., solubility, water/oil absorption), (ii) structural and rheological properties (e.g., viscosity, elasticity, gelation), and (iii) surface-related traits (e.g., emulsification, foaming) [72]. Proteins’ physical and chemical characteristics determine how they behave during processing, heating, storage, and consumption, which has a direct effect on the sensory qualities and quality of food [16,19,53,119]. Furthermore, in food formulation, the most important functional characteristics of protein are its solubility, ability to bind water and fat, gel-forming and rheological properties, emulsifying properties, foaming and whipping properties [6,53,72,90]. For example, faba beans’ solubility, foaming, emulsion, gelling, and water and/or oil-holding qualities can be used to create dairy and meat substitutes. In order to produce products with clearer labels, faba beans can also be used in place of various ingredients in food formulations [28,53]. Despite their advantages, the use of legume proteins depends on optimizing their functional properties for specific food applications [6]. Protein functionality is influenced by physicochemical and structural characteristics, which differ among leguminous crops and varieties [11,19]. Additionally, processing methods, processing conditions (e.g., sonification, irradiation, cooking, germination, etc.) enviromental factors (such as temperature, pH, pressure, ionic strength and/or the presence of other ingredients), can also affect the functional properties of protein and their applications in food industry [3,9,53,72]. For example, pea protein isolates often present challenges due to low foaming and emulsifying capacities, while the faba bean protein isolate shows potential as a sustainable ingredient but has limited functionality at neutral pH [93]. The legumin to vicilin ratio plays a significant role in affecting solubility and emulsifying properties [9]. Commercial extraction methods, particularly those involving high pH and temperature, can further reduce the functionality of pea protein, making it less effective in food applications compared to laboratory-prepared versions [41]. Additionally, selecting the right cultivars and optimised processing are also important to improve faba bean and pea proteins, ensuring they meet food industry and consumer expectations [47]. In general, the effective use of plant-based proteins as functional ingredients is essential to improve texture, stability, and consumer acceptance in food products [15]. Ongoing research is necessary to better understand the characteristics of these proteins and to enhance their applications in various food systems [120].

### 6.1. Solubility

Water solubility is a fundamental functional property of proteins in food systems [63]. Solubility is an important functional property of proteins which impacts the extent to which they can be utilised [9,53]. Protein solubility has a direct link to functional properties. It can be a good indicator of quality estimation in protein powers [22]. Protein solubility is essential for functional properties like gelation, foaming, and emulsification, which are critical in food applications [6,14,119]. Factors such as pH and salt presence influence solubility [7], and high solubility is often necessary for proteins to exhibit these functionalities effectively [6,9]. Plant proteins, particularly those from pulses, often have poor water solubility compared to animal proteins like whey or egg proteins. This limited solubility reduces their effectiveness as functional ingredients in food formulations, restricting their application in products requiring properties like emulsification, foaming, and gelation [6,9]. However, pulse proteins often exhibit better solubility around neutral pH compared to other plant proteins, such as cereal proteins. They are generally poorly soluble in the mildly acidic range, near the isoelectric points of the main protein fractions [9]. Above the isoelectric point, proteins carry a net negative charge, while they carry a net positive charge below their isoelectric point. The repulsive forces between similarly charged proteins is an important factor for protein solubilisation. Near the isoelectric point, the net charge is negligible and the proteins are prone to precipitation (typically between pH 4.0 and 5.0 for legume proteins) [9,11,53]. This generally narrows the range of suitable applications, and even near neutral pH (away from the isoelectric point), pulse proteins may be inadequately soluble in some cases. For instance, The solubility of 7 S and 11 S globulins from faba bean is dependent on the pH and ionic strength. Studies found that at low ionic strength, 7 S and 11 S globulins from faba bean had low to intermediate solubility at both pH 5 and 7. At a concentration of 0.5 M NaCl, the solubility of both 7 S and 11 S increased to around 90% at both pH 5 and 7 [105]. Similarly, a study found that pea protein isolates from different cultivars were more soluble at pH values of 1 and 7 than isolates from Kabuli and Desi chickpeas, as well as green and red lentils [106]. It has been suggested that commercial protein isolates often demonstrate relatively poor solubility compared with those produced at laboratory scale, attributable to denaturation during processing [9,11,53]. Nevertheless, poorly soluble proteins still find use in certain products such as meat analogs, baked goods, and protein bars, where solubility is less critical [11]. The solubility of proteins is determined by the balance between protein-protein and protein-solvent interactions, including repulsive and attractive forces [11,72,119]. These are influenced by factors such pH, salts, surface charge, and conditions in the environment [11]. Normally, native globular proteins fold in a way that exposes more hydrophilic regions at the surface and buries more hydrophobic regions at the centre. Solubility in a particular environment is determined by the structure of proteins and the ratio of polar and non-polar groups exposed to the surface [9]. Repulsion due to similarly charged proteins promotes solubility, whereas hydrophobic interactions between proteins promotes aggregation and lower solubility. Both intrinsic and extrinsic environmental factors influence solubility [1,9]. Aside from the protein’s intrinsic structural properties, the dispersion preparation method/conditions (e.g., homogenisation vs stirring) can have a major impact on solubility values that should not be overlooked [9]. In addition, the protein extraction method also affects its solubility. For example, Pea protein isolates extracted by alkaline extraction with isoelectric precipitation resulted in a solubility of 90% at pH =1, but only 29% at pH = 3, whilst ultrafiltration extracted isolates had solubilities of 60% at pH = 1, and 56% at pH = 3 [106]. Pea protein isolates extracted at a higher pH (pH = 9.5) contained more poor soluble protein aggregates at both acidic and neutral pH than those extracted at a lower pH (pH = 8.5). This could be because higher alkaline pH promotes protein aggregation, which is caused by things like decreased repulsive forces, altered protein conformation, and increased cross-linking between the proteins. The researchers ascribed the reduced solubility of the isolates from alkaline extraction to protein–protein hydrophobic interactions with isoelectric precipitation and micellization [106]. Solubility in water is essential for proteins in applications like beverages, emulsions, and foams used in products such as salad dressings, whipped toppings, and nutritional drinks [11]. The interaction of proteins in solution is crucial for stabilising foams and emulsions, and their effectiveness can be influenced by intrinsic factors (e.g., protein composition, overall net charge and isoelectric point) and extrinsic factors (e.g., temperature, pressure and pH) [1,9]. Various techniques, including physical, chemical, and enzymatic approaches, are being explored to enhance solubility and thereby expand the use of pulse proteins in food products [11,41]. Enzymatic hydrolysis, by reducing the molecular weight and exposing hydrophobic regions and ionizable groups, is a strategy used to improve solubility. This method has proven effective for improving the application potential of pulse proteins by modifying their structural properties [9]. For example, high intensity ultra-sonication reduces the particle size of protein dispersions, increasing water solubility due to a larger interaction area between protein and water molecules. Faba bean proteins hydrolysates with lower molecular weights exhibited higher solubility because smaller peptides produced by hydrolysis can form stronger hydrogen bonds with water and become more soluble. Hydrolysis of faba bean proteins by alcalase enzyme improves the solubility by 8–10% at pH 8 [105].

### 6.2. Water Holding Capacity (WHC)

The water holding capacity, sometimes also expressed as water binding capacity or water absorption capacity (g of water/g of protein) refers to the ability to avoid water releasing from the proteins three-dimensional structure [119,121]. Protein-water interactions affect various functional characteristics such solubility, foamability, emulsification, and gelation (little is known about the nature of “bound” water and how it relates to protein solubility). WHC is very important when it comes to dry powder products, like protein isolate or concentrate, and it should be taken into consideration [119]. Water binding is a critical functional property of pulse proteins that determines their ability to absorb and retain water during food processing and storage [121]. This characteristic plays an important role in determining the texture, freshness, mouthfeel, juiciness, and overall sensory attributes, and stability of food products such as baked goods, soups, doughs plant-based meats, protein-rich snacks and emulsions [41,122]. WHC influence the textural and sensory properties of foods in various ways. The WHC must be considered when designing plant-based hydrocolloid food products to ensure they retain moisture during production and shelf-life. Plant-based yoghurts must be able to retain water over time to avoid phase separation, which is considered unappealing by consumers [114]. It is an important property to consider when producing protein-based gels [121]. It also serves as an indirect reflection of the strength and extent of intermolecular interactions within the gel matrix, particularly hydrogen bonding and hydrophilic interactions [122]. The number of polar groups, surface hydrophilicity, and particle size all affect the rate at which protein powders absorb water. Protein intrinsic characteristics, concentration, ionic strength, pH, temperature, and process conditions all have an impact on a proteins WHC [10]. For instance, WHC rises at low salt concentrations because salt ions in the solution improve water binding to protein. Furthermore, the water-binding properties of food are also influenced by additional food components like lipids, salts, and hydrophilic polysaccharides [119]. The WHC is also lowest at protein’s isoelectric pH since protein-protein interactions are at their peak. Because salt ions bind water to proteins, WHC increases at low salt concentrations [106]. WHC of faba bean proteins is show as a function of pH as it increased with pH as reported by Żmudziński et al. [16] that at pH 4, 5, 6, 8, water holding capacities were 2.2, 1.9, 2.5, and 3.4 respectively. This pH-dependent behavior suggests that faba bean protein isolates (FBPI) water-binding capabilities can be optimized by adjusting the pH environment, which is crucial for tailoring its application in specific food formulations [16]. Water holding capacity of faba bean protein isolates extracted with ultrasound and enzymatic hydrolysis was measured to be between 2.1 and 4.7 g/g [105]. Pea protein isolates produced using various techniques may have varying WHCs. Researchers that studied various pea cultivars found that protein isolates isolated via micellization had higher WHC values. They linked this to the exposure of side chains and polar groups, which increases hydrogen bonding. However, pea proteins extracted using the alkaline extraction with isoelectric precipitation method from five pea genotypes have a greater WHC (3.9–4.8 g/g). Additionally, WHC values for pea protein isolate (PPI) vary significantly, ranging from 1.2 to 6.0 g water/g protein, depending on the genotype and extraction method employed [112]. On the other hand, a study reported WHC values of 1.93 g water/g dry seed for pea protein isolate and 1.87 g water/g dry seed for faba bean protein isolate, indicating a slight advantage for pea proteins in water retention [123]. Another investigation highlighted that pea protein isolate achieved WHC values ranging from 3.2 to 3.6 g/g when extracted using micellar precipitation, surpassing the 2.4 to 2.6 g/g observed with alkali extraction-isoelectric precipitation methods [124]. Consequently, depending on the cultivars, different extraction techniques may have varying effects on the WHC of pea proteins [106]. The differences in WHC between PPI and FBPI can be attributed to their distinct protein compositions and structures [124]. PPI contains higher levels of vicilin, a protein known for its superior water-binding properties, while FBPI has a higher proportion of legumin, which may contribute to its comparatively lower WHC [123]. Additionally, the presence of smaller starch fragments in PPI enhances its ability to retain water [123,124]. The differences in WHC between PPI and FBPI can be attributed to their distinct protein compositions and structures [124]. PPI contains higher levels of vicilin, a protein known for its superior water-binding properties, while FBPI has a higher proportion of legumin, which may contribute to its comparatively lower WHC [123]. Additionally, the presence of smaller starch fragments in PPI enhances its ability to retain water [123,124].

### 6.3. Oil Binding Capacity (OBC)

Oil binding capacity, also known as oil or fat holding capacity (OHC or FHC) or oil or fat absorption capacity (OAC or FAC), is the capacity of proteins to interact with lipid molecules and absorb and retain oil. Because it affects the distribution and emulsification of oil, it is crucial in food systems [119,121]. This interaction is facilitated by the binding of lipid aliphatic chains to nonpolar side chains of amino acid residues, with proteins possessing higher hydrophobicity showing greater oil affinity [106]. OHC of proteins is related to the texture, mouthfeel and flavour retention of products. Oil-holding capacity is required in ground meals, meat analogues, extenders, beverages, and salad dressings in which oil contributes to the texture and mouthfeel of food products. OHC usually increases with denaturation, as the proteins’ hydrophilic core is exposed, thus favouring oil-binding [106,114]. OHC is influenced by protein sources, molecular structures, processing conditions, droplet size, and the distribution and stability of lipids. OHC can be impacted by structural alterations carried on by temperature and pH changes as well as modifications in protein processing, which can affect the overall quality and texture of food products [106]. The effectiveness of OHC, which is essential for emulsion stability and overall sensory experience in a variety of food formulations, is also significantly influenced by the size of oil droplets and the stability of lipid-protein complexes [106]. Hydrophobic, electrostatic, and hydrogen bonding constitute protein-lipid interactions. Proteins with a higher OBC have more hydrophobic regions because hydrophobic bonding is essential for the stability of protein-lipid complexes. The type of oil and its distribution, the source and concentration of proteins, and the processing conditions all affect how well food proteins absorb oil. The size of the particles can also affect oil absorption in protein powders. Typically, more oil absorption capacity is seen when the particle size decreases [119]. Molecular weight of faba bean protein fractions also affects the oil absorption capacity as it decreases with molecular weight of the protein isolates. Smaller peptides are less proficient in entrapping the oils [105]. It was shown that isoelectric precipitation produced a lower oil holding capacity (0.67 g oil/g protein), whereas ultrafiltration-diafiltration precipitation produced a pea protein with a similar oil holding capacity (2.21 g oil/g protein) to salt extraction (2.16 g oil/g protein) [106].

### 6.4. Interfacial Properties

Proteins act as natural ingredients in food emulsions due to their amphiphilic nature and their ability to form interfacial films at the oil-water interface. The adsorption of protein at interfaces generally involves three main steps. The first protein migrates from the bulk phase to the interface. Thereafter, proteins adsorb at the interface, resulting in structural changes. Finally, an interfacial protein network is formed through intermolecular interactions and multilayer structures [77]. The emulsifying properties of proteins are influenced by interfacial properties, functional attributes (e.g., solubility) and molecular structure (e.g., flexibility and rigidity). Surface hydrophobicity affects the adsorption of proteins at the oil-water interface, while solubility determines the rate at which protein molecules migrate to this interface [121]. The use of plant proteins as emulsifiers is particularly appealing to the food and beverage industries due to their safety, nutritional value, and functional properties. For example, pea protein, with its excellent physicochemical characteristics such as high water and oil absorption, superior gelation capabilities, and gel clarity, offers a novel plant-based option for functional food formulations [53]. Pea protein also serves as a binder, emulsifier, stabiliser, or extender in various food applications [122]. Although proteins exhibit good emulsifying capacity, emulsions stabilised by proteins are sensitive to environmental factors such as pH, ionic strength, and thermal processing [53]. Pea protein has been successfully employed as an emulsifier in liquid emulsions and spray- dried emulsions for microencapsulating oils. Studies have shown that pea protein reduces interfacial tension between water and oil, stabilising emulsions by forming a rigid membrane at the oil-water interface. Its high surface-active properties enhance its ability to stabilise food emulsions, and key processing conditions for optimising this capacity have been characterised [53]. In addition to its emulsifying abilities, the foam-forming capacity of protein is crucial for products like cakes, soufflés, whipped toppings, and fudges. Notably, pea protein outperforms soybean protein as an emulsifier and foaming agent at neutral pH [53].

### 6.5. Emulsification Properties

An emulsion is made up of two immiscible liquids (mainly oil and water), one of which is distributed throughout the continuous phase of another liquid as droplets [114]. Food emulsions are often composed of water and lipids, hence the two main categories of emulsions are “water-in-oil (W/O)” and “oil-in-water (O/W)”. The emulsions intrinsic thermodynamic instability system results from the interfacial tension that exists at the interface between two liquid phases [119]. The interfacial surface tension between these two liquids causes a thermodynamically unstable system inside the emulsion requiring stabilization by emulsifiers. Once the emulsion has been formed, a good emulsifier should also play a critical role in preventing various occurrences that will destabilize the emulsion, including coalescence, flocculation, sedimentation and creaming [63]. This effect is commonly measured over emulsifying ability and capacity [114]. The demand for plant proteins as natural emulsifiers is growing due to “clean labelling” trends and the shift away from animal-based ingredients [3]. Pulse proteins can act as plant-based emulsifiers due to their surface activity, which allows these proteins to stabilise emulsions in plant-based foods, such as dairy analogues and dressings [114]. Proteins act as effective emulsifiers by reducing the interfacial tension between immiscible liquids like oil and water, aided by their amphiphilic properties and structural rearrangements at the interface [9,119]. They do this by adsorbing to oil-water interface and stabilising droplets [106]. Emulsion stability depends on protein solubility, rapid adsorption, formation of a viscoelastic layer, and electrostatic repulsion to prevent droplets aggregation [125]. Pulse proteins, derived from legumes such as peas and lentils, are particularly valued for stabilizing emulsions, enhancing texture, and preventing phase separation, offering a sustainable alternative to traditional emulsifiers [3,9]. Pea protein exhibits excellent emulsifying properties, particularly for O/W emulsions [53]. Chemical modifications, such as acetylation or succinylation of reactive lysine residues, enhance its emulsification performance. Emulsification capacity is pH-dependent, being lower near the isoelectric point and improving above pH 7. The impact of pH on the stability of protein isolate-stabilized oil-in-water (O/W; 10/90 wt%) emulsions revealed that emulsion stability significantly improved as pH values deviated from the isoelectric point (pI = 4.5). Protein isolates from peas, chickpeas, and lentils have been shown to have emulsifying properties, including droplet size dispersion, flocculation, coalescence, and creaming [114]. Hydrolysis with enzymes, such as chymosin, further enhances foaming and emulsifying properties, with solubility positively correlating with the emulsifying capacity [53]. The emulsifying properties of proteins depend on characteristics such as water solubility, surface charge, hydrophobicity, shape, size, and structural flexibility [63,106]. In the case of pea protein, the extraction method significantly influences its emulsifying capacity. The extraction methods such as ultrasound-assisted alkali extraction and ultrafiltration enhance the emulsifying activity index and emulsifying stability index of pea protein isolate [106]. This can be attributed by improving extraction efficiency, modifying protein structure, reducing particle size, and increasing surface hydrophobicity. Pea protein isolates obtained via alkaline isoelectric precipitation also show superior emulsifying properties compared to those extracted with sodium-based methods, with emulsifying activity index and emulsifying stability index positively correlated with solubility and surface charge, and negatively with hydrophobicity [106]. Production methods like isoelectric precipitation or salt extraction affect the functionality of the pea protein isolate by altering the globulin/albumin or legumin/vicilin ratios and physicochemical properties [53]. The pea protein isolate produced via isoelectric precipitation has higher surface charge and solubility compared to salt-extracted pea protein isolate. Emulsifying abilities vary with pH, being highest at pH 3.0 and lowest at pH 5.0. Alkaline treatment improves interfacial properties, steric hindrance, and oxidation resistance in emulsions [53]. Heat treatments, such as pasteurisation and sterilisation, influence droplet size, flocculation, and creaming stability in pea protein-stabilized emulsions by inducing inter-droplet hydrophobic interactions [53]. Overall, field pea proteins are effective stabilisers of oil-water interfaces due to their surface-active properties [122]. In the same way, the protein from faba beans forms a stable emulsion. Researchers found that the emulsifying capacity of vicilin was significantly more effective than legumin at pH 7 thereby concluding the faster adsorption rate of the former at the oil-water interface [105]. Modification of faba bean protein by moderate hydrolysis using alcalase enzyme increases the surface hydrophobicity which could be ascribed to exposing more hydrophobic groups and the emulsions prepared by modified protein results in higher physical and oxidative stability. After suitable modifications, the emulsifying activity and emulsion stability of faba bean proteins can be improved and that could be a capable plant based emulsifier in food products [105]. All proteins’ surfaces can become more hydrophobic through high pressure processing or heat treatments, although solubility is reduced. Remarkably, neither heat treatment nor high-pressure processing changed the faba bean protein’s capacity to emulsify. The development of aggregates during these treatments may have countered the advantages in emulsification activity that were anticipated due to the increase in hydrophobic character and structural flexibility after protein denaturation. Nonetheless, the emulsion stability of heat-treated faba bean protein concentrate was significantly higher than that of untreated and high pressure-treated ones [63]. These results show that conventional modification hardly enhances the faba bean protein’s capacity for emulsification. Therefore, new approaches should be investigated in the near future or an optimization of parameters should be found [63]. Through innovative extraction and processing methods, ongoing research aims to better understand and enhance the emulsification properties of pulse proteins. The development of more stable and useful emulsified products may result from research on the interactions between pulse proteins and other food ingredients, including lipids and polysaccharides. Furthermore, in the rapidly growing plant-based food industry, knowing the structure-function relationships in pulse proteins will help formulate components specifically suited for specific applications [22].

### 6.6. Foaming Properties

A foam is defined as a two-phase mixture in which the gaseous phase is surrounded in a continuous phase (liquid or solid). However, foam is a thermodynamically unstable system and will easily collapse unless stabilised. Some proteins are excellent foaming agents [119]. Foaming and emulsifying properties are critical for the production of specific food products. Plant proteins, optimised through protein fractionation, can provide these functionalities [94]. Foams are dispersions of gas bubbles in continuous phase, stabilised by proteins, which act as surfactants, forming a viscoelastic layer at the air-water interface to reduce interfacial tension, similar to emulsions [9]. Foaming properties are vital in food systems, contributing to the texture, volume, structure and lightness of products such as whipped toppings, mousses, bread, meringue, cream and cakes [10,16,106]. The key indicators used to characterize the foaming properties of proteins are foaming capacity and foaming stability [106]. Stable foams are essential for applications like ice cream, cakes, and whipped toppings. In bakery products, effective foaming enhances batter aeration, resulting in lighter textures and improved sensory qualities [22]. Foaming capacity and stability could be affected by the solubility, molecular flexibility, conformation and protein molecular weight, and bubble size. Protein surface-hydrophobicity and molecular weight mainly influence the initial rate of protein adsorption at air-water interface, a higher surface hydrophobicity and lower molecular weight help protein molecules to quickly diffuse to the air-water interface, which led to encapsulating air particles and increasing foaming capacity of proteins [9,105,119,121]. A cohesive viscoelastic protein film at the air-water interface is vital for stability, while solubility, concentration, and surface hydrophobicity also play roles. Proteins difficult to denature typically exhibit poor foaming capacity [31,121]. Key factors affecting foaming include adsorption speed, protein rearrangement, cohesive film formation, protein concentration and fraction, solubility, pH, ionic strength, method of protein extraction and surface hydrophobicity [9,10,106]. The solubility, surface flexibility, and hydrophobicity of proteins are the main determinants of an efficient foam formation [119]. A cohesive viscoelastic protein film at the air-water interface is vital for stability, while solubility, concentration, and surface hydrophobicity also play roles. Proteins difficult to denature typically exhibit poor foaming capacity [31,121]. Foaming capacity of faba bean protein varies according to pH, at pH 4, 5, 6, 7 it is 25%, 31%, 50%, and 67% respectively. Lipid content has negative effects on foaming ability of proteins as lipids can behave as antifoaming agents [105]. The water-soluble fraction of pea protein was shown to have a significantly higher foaming capability at pH values of 4 and 7, but not at pH 9, when compared to other fractions such commercial pea protein isolate, ethanol-soluble, alkaline-soluble, and salt-soluble. Commercial pea protein concentrate was shown to have a foaming capacity similar to that of commercial soy protein isolate, although its foaming stability was better than the latter [106]. Pulse proteins, as plant-based foaming agents, offer a vegan and allergen-free alternative to egg whites, aligning with market trends [22]. Their foaming ability relies on rapid adsorption to the air-water interface, surface tension reduction, and forming a stabilizing viscoelastic film around air bubbles that stabilises the foam [119]. Protein solubility and surface tension are critical, with isolates outperforming concentrates due to higher protein purity [16,31]. The foamability of plant proteins such as faba bean and pea proteins can be improved at an alkaline pH. This condition increases the surface hydrophobicity of the proteins, enhancing air entrapment [31]. Enzymatic hydrolysis can improve can also foaming properties [9,106].

### 6.7. Gelation Properties

Gelation properties refer to the ability of a protein to form a semi-solid three-dimensional network by unfolding and aggregating under conditions like temperature, pH, or ionic strength [9,10]. Protein gelation involves the formation of a three-dimensional network through interactions such as protein-water, protein-fat, and protein-protein, influenced by factors like concentration, temperature, pH, ionic strength, additives, and enzymes [53,119]. Protein gels may hold water, fat, sugars, and other constituents. The gel formation process is based on a (partial) protein denaturation with the resultant conformational changes (access to active sites), followed by interactions, aggregation and final gelation [119]. This property is crucial for the texture of various food products, including plant-based meat alternatives, dairy substitutes (cheese, yoghurt), tofu, and desserts [121]. Pulse proteins exhibit gelation capabilities, but often form weaker gels compared to soy proteins due to fewer sulfhydryl groups and disulfide bonds [9]. Protein gels are stabilised by both covalent and non-covalent (electrostatic, hydrogen bonds, hydrophobic) interactions and require a critical concentration for gelation to occur [9,119]. Advanced techniques like heating-cooling, acid or salt treatment, cross-linking agents (such as transglutaminase, polysaccharides, and polyphenols), and microbial fermentation can enhance gelation [121]. These gels can also encapsulate unstable compounds like vitamin E and β-carotene for improved stability. The gelation process is influenced by physicochemical properties such as particle size, molecular structure, surface hydrophobicity, and intermolecular interactions [121]. For instance, depending on how much charge the original protein carries, globular proteins those in faba bean protein isolates typically produce two types of gels. Proteins from faba beans typically have an isoelectric point of 5.0 to 5.5 when NaCl is added. Most favourable percentage for gel formation of the faba bean protein is 15–15.5%. Regardless of the extraction technique or the addition of sodium chloride, coarse particle gels may form at pH 5 while finer gels require pH 7. For the gels prepared from alkaline protein extracts of faba beans, significant relationships between compressive stress and strain at fracture were observed [105]. Despite the challenges in replicating the textures of traditional products, pulse proteins play a functional role in the development of innovative plant-based alternatives [121]. The heat-induced gelation of the pea protein is influenced by extraction methods, temperature, pH, salt, and composition, which affect the formation of aggregates of soluble proteins that can form gel networks [53]. According to a study on the gelation properties of pea proteins, pH has a greater impact on functional properties than ionic strength. The gels that were the stiffest were produced at 0.6 M NaCl and pH = 4.5. An entangled solution rather than a gel was formed at a pH of 9 and ionic strengths of 0.9 M and 1.5 M NaCl_4_ [106]. At low pH 4 values, the ionic strength had a greater impact on pea protein gelation than at alkaline or neutral pH values. If the protein concentration is high enough, denaturation of the protein can lead to the development of aggregates and then gels. It was discovered that denaturation and heat-set gelation of pea protein are influenced by pH and ionic strength. Additionally, It was demonstrated that NaCl retarded denaturation, and the stiffest gels could form at pH values below 6 and 0.3 M NaCl [106]. Ultrafiltration and diafiltration enhance the functionality of these aggregates in cold-set gels. The denaturation temperature of pea proteins increases with a higher legumin content, and the disulfide-linked legumin subunits aggregate between 75–85 °C [53].

Cold-set gelation offers better control over protein aggregation, influencing acid-gel strength [53,119]. While legumin thermal aggregates reduce solubility and impair acid gelation, heating rates do not affect gel formation. However, slower cooling rates impact gel development in pea protein samples. These findings highlight processing parameters critical role in optimising gelation properties for food applications [53]. However, it has poor gelling properties due to limited solubility, low cysteine content for disulfide bonding, and susceptibility to thermal denaturation. Additionally, pea protein requires high temperatures and concentrations to form gels, which limits its application in food products. High viscosity at concentrations above 10% further restricts its use in emulsions [53,122]. Researchers have looked to polysaccharides as natural texture enhancers and efficient pea protein gel modifiers to overcome these limits of pea protein in gelation properties. During heat-induced gelation, polysaccharides may interact with proteins via covalent bonding, hydrogen bonding, hydrophobic contacts, and electrostatic associative interactions [122]. Heat is a common method for inducing gelation in proteins like pea and faba bean, which have a denaturation temperature below 95 °C [31]. Both proteins can form heat-induced gels in acidic conditions, similar to soy protein. However, the pea protein forms weaker and less elastic gels compared to soy, limiting its use in applications requiring firmer gels [31]. While heat is the most widely used method for protein gelation, alternatives such as pressure, pH shifting, and enzyme crosslinking are also possible, though research on these methods for pea, oat, and faba bean proteins is limited [31]. However, Faba bean protein exhibits similar rheological properties and gel strength to soy protein during heating and cooling [31].

### 6.8. Thermal Properties

Protein thermal denaturation helps understand their structure-functional potential. When proteins are subjected to changes in temperature (e.g., during processing), heat exchange (endothermic or exothermic) will occur due to various physical or chemical changes [119]. The thermal behaviour of pulse proteins, including their stability and structural changes under heat, significantly impacts their functionality in food applications [119,121]. Heating causes denaturation, gelation, and structural alterations, affecting solubility, emulsification, texture, and gelation properties [10,22]. These thermal properties are crucial for optimising high-temperature processes like baking, cooking, extrusion, and pasteurisation, allowing the formation of gels, meat-like textures in plant-based meat alternatives or analogues, and high-protein snacks. Furthermore, heat treatment improves digestibility and reduces anti-nutritional factors present in raw pulses. Thermal properties also help maintain protein integrity in baked goods, contributing to their texture and volume. Protein composition, extraction process, moisture level, and presence of additional food ingredients are some of the variables that affect the thermal characteristics of pulse proteins. The thermal stability of different pulses varies because of variations in the composition and structure of proteins. Proteins recovered by wet fractionation, for example, are typically more denatured and may react differently to heat than proteins obtained by dry fractionation [22]. During heating, the way pulse proteins interact with other food ingredients like fats and carbohydrates can also impact their functionality and thermal stability. Research shows that ultrasound treatment and acylation (acetylation and succinylation) improve the thermal stability of plant proteins [121]. Enzymatic treatments can further enhance functional properties by breaking down anti-nutritional factors and modifying the protein structure [126]. Current studies focus on improving the stability and functionality of pulse proteins through advanced processing and modifications. Investigating their thermal interactions with ingredients like polysaccharides and emulsifiers may lead to better formulations for high-temperature food processes. Understanding thermal denaturation is the key to optimising pulse proteins for innovative plant-based products, including extruded snacks and protein-enriched beverages [22].

## 7. Structural Modification Techniques for Improvement of Functional Properties

**Figure 3:**
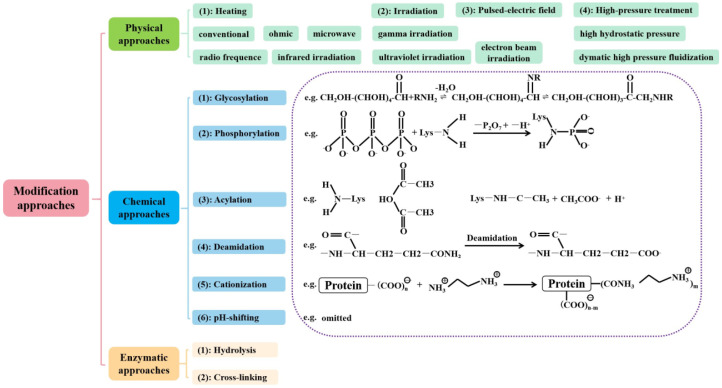
Physical, chemical, and enzymatic methods for the modification of plant-based proteins [3].

Pulse proteins are valuable in enhancing the nutritional value and functionality of food systems, but often require modifications to address challenges related to nutrition, sensory attributes, and functionality [3,90,127]. The term “protein modification” refers to the process of altering the molecular structure or a few chemical groups of a protein by special methods for the purpose of their techno-functionality and bioactivity improvement [128]. The differing protein structure will influence the protein functionality in a variety of ways. It is for this relationship between a protein’s structure and functionality that causes the target of protein modification to be protein structural changes [63]. Modification of pulse proteins provides the opportunity to make them multi-functional ingredients for food systems by changing their physicochemical properties and addressing their limitations [63,128]. Native pulse proteins may contain antinutrients, undesirable flavours (e.g., beany flavour) [90]. Protein modification involves altering amino acid residues and polypeptide chains to improve spatial structure and functionality [127]. Modification techniques include physical, chemical, enzymatic, combined, and bioengineering methods (Figure 3) [63,90,108,127]. These processes thermodynamically alter protein structure and conformation, enhancing properties like solubility, emulsification, and sensory appeal for specific applications [53,90,108]. Such advances enable the broader use of pulse proteins in plant-based and alternative food products [90]. Processing can change the nutritional content of the ingredients overall, modify the structure and conformation of the protein and other components, or alter the overall nutritional composition after processing steps [4]. Pulse proteins are promising substitutes for animal proteins because of their nutritional and functional benefits. However, their application in food processing is limited by the diverse properties of different pulses [5]. The functional characteristics of proteins are heavily influenced by their three-dimensional structures, which pose challenges to plant proteins in replicating the functionalities of animal proteins [5]. This includes difficulties in mimicking the fibrous structure of muscle tissue or the specific structural properties of dairy proteins, such as caseins, which are critical to the texture of cheese and yoghurt [3,9]. Additionally, pulse proteins tend to have poor solubility in mildly acidic conditions, particularly near their isoelectric point, which hinders their application in mildly acidic food systems [9]. To address these issues, studies have demonstrated that complexing plant proteins with other compounds can enhance their functional properties, including gelation, emulsification, and solubility [9]. To enhance functionality in food formulations, additional ingredients are often incorporated to achieve desired product characteristics like softness, juiciness, and stability [108]. Protein-protein interactions, driven by hydrogen bonding, electrostatic forces, hydrophobic interactions, and disulfide bonds, play a key role in improving functionality. Factors like pH, temperature, ionic strength, and protein concentration are critical in facilitating these interactions [3,9].

Hybrid protein systems show promise for developing innovative, synergistic functionalities, allowing plant proteins to serve as effective alternatives to animal proteins. These systems leverage structural versatility and amphiphilic properties to expand plant protein applications in food systems [3]. The functional traits, such as solubility, water and oil absorption capacities, gelation, and surface activity, ultimately dictate the behaviour and performance of proteins in food products [120]. More attention has recently been paid to the structural and functional characteristics of pea protein isolates [14]. Pea protein forms highly viscous solutions at high concentrations, limiting its functional properties. Enzymatic treatments, favoured over chemical modifications, are widely used to address this issue due to their specificity, mild conditions, and minimal side reactions [53]. Modifications of pulse proteins, including enzymatic and physical treatments, are necessary to unlock their full potential as alternatives to animal proteins in food products. These techniques help address the key limitations of pulse proteins and improve their functionality for diverse applications.

### 7.1. Physical Modification

Physical alterations occur when pressures and/or energy are applied to plant protein without the addition of other substances. Physical methods of protein modification primarily use physical forces to enact changes upon the protein structure, therefore altering the protein functionality [129]. Physical modification is very popular in the food industry. These methods may result in size reduction, size redistribution, unfolding, depolymerization, and irreversible denaturation of plant proteins, among other alterations to their macrostructure and microstructure [129,130]. Physical modification techniques, including extrusion, heating, ultrasonification, pulsed electric fields, high-pressure homogenization, and microwave treatments, can enhance pulse proteins functional properties by inducing structural modifications without altering their chemical composition [22,103,127,130]. Physical alterations, such as partial denaturation and controlled aggregation, enhance properties like heat stability, solubility, and foaming ability, helping bridge the functionality gap between plant and animal proteins [6,127]. These techniques are cost-effective, no chemical reagent involved, require minimal processing time, and result in negligible nutritional loss, making them valuable tools to improve the performance of plant-based protein ingredients in food systems [127,131]. However, the success of physical protein modification is contingent upon the processing parameters, such as treatment time, temperature, pressure, etc. Additionally, it is critical to be aware that different plant proteins (whether they be different fractionations or come from a different source) may not react equivalently to the same treatment conditions. Therefore, it is important to understand the modification process and the protein in question so one can optimise the treatment [63].

#### 7.1.1. Heat Treatment

Heat treatment is a widely used physical modification technique to enhance the functional properties of pulse proteins. By inducing partial denaturation and unfolding, heat causes structural changes that influence solubility, emulsifying capacity, gelation, and foaming properties [90,127,132,133]. During denaturation, proteins lose their secondary and tertiary structures, exposing hydrophobic cores. Subsequent aggregation occurs through hydrophobic interactions, hydrogen bonds, and disulfide (S-S)/sulfhydryl bonding [90,127,132,133]. While heat treatment can enhance gelation through increased protein-protein interactions, excessive aggregation may reduce solubility. Mild heating promotes the formation of disulfide bonds, leading to insoluble aggregates. Controlled heat treatments, with careful adjustment of temperature, time, pH, ionic conditions, and protein type, can optimise structural modifications to achieve desired functionality in protein ingredients [90]. Excessive heating can negatively impact protein functionality by promoting aggregation, which reduces solubility and diminishes performance in food systems. Over-denaturation may also degrade essential amino acids, lowering the proteins nutritional quality [10]. However, in some cases, severe heat treatment can enhance solubility by breaking disulfide linkages, showcasing the nuanced effects of heating on protein structure and functionality [127]. Pulses and soy proteins also showed this effect. Heat has the ability to alter other functional characteristics including colour, flavour, and aroma. It is noteworthy that boiling soy protein solutions improved their digestion and decreased their raw or beany taste [127]. According to Zhang et al. [127], certain heat treatments, including steam cooking, improve nutritional quality by making proteins more digestible and may cause caramelization, which adds flavours that are sweet and caramel-like. However, too much caramelization might result in bitterness, which emphasizes the importance of carefully weighing the type of protein ingredient, heating temperature and duration, and environmental factors [127]. In work [116] investigated the effect of dry-heat treatment on the properties of faba bean protein concentrate. Faba bean protein concentrate was dry-heated at temperatures from 75 to 175 °C, which resulted in higher water-holding capacity and less soluble protein. These changes were due to partial denaturation of protein, changing the structure of the protein, and exposing hydrophobic sites. This led to protein aggregation, as observed by light microscopy [90]. Only non-covalent bonds caused the decrease of solubility of dry-heated faba bean protein concentrate. Careful control of the heating and cooling rates enables maximum gel strength for heat-induced pulse protein gels. This enables the utilisation of pulse protein as an additive in the meat food industry [90]. As studies reported improved emulsifying properties in pea protein isolates with heat treatments. The heat treatment of the pea proteins at 95 °C for 30 min increased the extent of protein aggregation and had increased vicilin and legumin B in the adsorbed protein layer in the emulsion [90]. Proteins solubility is typically reduced upon heating due to aggregation. Protein deformation properties depend primarily on the number of disulfide bridges and β-sheet content [134]. For example, the high content of disulfide bridges in native 11S fraction of lupin protein isolates prevented molecular reconfigurations upon heat treatment. The proteins with a greater proportion of β-sheets were thermally more stable, need a higher temperature for gel formation, and produce stronger gels due to greater intermolecular interactions between them [90]. Heat treatment could prevent legume protein from being affected by microorganisms and anti-nutritional compounds like protease inhibitors [128]. In addition, heat treatment could improve the functional properties of legume protein by changing its structure, while its functional properties depend on the degree of protein denaturation [76].

#### 7.1.2. High-Pressure Processing (HPP)

High-pressure homogenisation (HPH) is a novel non-thermal processing method for modifying the functional characteristics of proteins. Compared to other structure-modifying techniques like ultrasonication and pH adjustment, HPH is better suited for large-scale industrial applications due to its continuous operation advantage [66,130]. The physicochemical and structural changes that proteins undergo under the mechanical stresses and short-term temperature impacts produced by HPH lead to changes of the proteins solubility, emulsifying ability, and foaming stability [66,130]. High-pressure processing is increasingly used to modify plant protein structures, enhancing their technological properties [83,108,135]. Applying high pressures (100–600 MPa) disrupts hydrogen bonds, ionic interactions, and hydrophobic interactions, leading to protein denaturation and the formation of new protein-protein or protein-water interactions. This alters the secondary and tertiary structures of pulse proteins, improving solubility, emulsifying activity, and gelation capacity while preserving their nutritional value [103,135]. HPP induces conformational changes by compressing protein molecules, breaking non-covalent bonds, and forming new interactions. The extent of these changes depends on the characteristics of specific protein such as pH and ionic strength, pressure level, temperature, and duration of treatment, making it a versatile tool for tailoring protein functionality [83,90]. Used high pressure technique (600 MPa, 5 min, 5 °C, 12% (*w*/*w*) plant protein gel) [136] on pea and faba bean protein isolates to create an alternative structuring strategy for plant-based yogurts. High pressure formed viscoelastic gels for all plant protein samples with comparable gel strength to commercial dairy yogurts. The high pressure homogenisation technique has shown an effect on the aggregation of faba bean globulin proteins and subsequently on their functional properties [90]. This method modulates the hydrophobic interactions among protein aggregates, leading to the dissociation of large protein aggregates (>1 μm) into soluble supramolecular aggregates. Higher surface hydrophobicity and the dissociation of insoluble protein aggregates led to faster adsorption of protein at the air-water interface. High pressure homogenised faba bean proteins have shown improved foaming properties and decreased emulsifying properties [90]. HPP has been shown to enhance the gelation properties of pea proteins, resulting in stronger, more cohesive gels compared to untreated proteins [137]. Faba bean proteins treated with HPP at 400 MPa showed improved solubility and emulsification capacity due to partial denaturation and enhanced surface activity [31]. Pea protein concentrates subjected to HPP demonstrated improved foaming capacity and stability, making them suitable for bakery applications [22,31]. In pea proteins, HPP was shown to have the ability to increase solubility and oil holding capacity, while on faba bean proteins, HPP improves their solubility and interfacial properties, but the emulsifying capacity was not affected [108].

#### 7.1.3. Heat with Shear Treatment (Extrusion)

Extrusion is a high-temperature, high-shear, short-time process that modifies the molecular structure of proteins by applying mechanical and thermal energy [31,66,138]. During extrusion, proteins are subjected to thermal and mechanical stresses, including heating, pressure, and shearing, which induce the unfolding of native globular proteins [66]. This process aligns protein molecules in the direction of flow, forming a fibrous structure supported by intermolecular bonds and aggregations. The result is a continuous, viscoelastic mass, making extrusion a key technique for developing textured plant-based proteins with enhanced structural and functional properties [31,128]. This viscoelastic mass can be aligned, crosslinked, reformed, and converted into an expandable structure that has a meat-like texture. Extrusion can be used to produce pre-cooked and dehydrated foods and has the ability to develop products with better nutritional, functional and sensory characteristics. Recent research focuses on using extrusion technology to produce legume-based meat extenders and meat analogues. Extrusion alters the free sulfhydryl content, particle size, and protein structures (secondary and tertiary) of legume protein isolates, impacting their functional properties [66]. High moisture and low moisture extrusion methods can be used based on final product requirements [126,138]. High moisture extrusion cooking is commonly used to produce meat analogs by producing desirable texture/fibrous structure to vegetable proteins at a high temperature needed to denature vegetable proteins by the shearing force of screw rotation inside the barrel leading to the unfolding of peptide bonds and the destruction of the three-dimensional structure of proteins resulting in the formation of cross-links of hydrogen, amide, and disulfide bonds between denatured proteins [126]. Low-moisture (<40% moisture content) extrusion technology can be used to produce plant-based extrudates that have fibrous structures and that give good functional properties including high water holding capacity and rehydration ratios [139]. The extrusion process enhances the native protein structure by denaturing it, which exposes specific amino acids and reduces heat-sensitive compounds like phytic acid, trypsin inhibitors, and tannins. This is achieved through the combined effects of thermal treatment and mechanical shear from the screw speed [138,139]. This also leads to an increase in protein and starch digestibility, exploitable to produce ready-to-eat products [66,90]. Feed rate, cooking temperature, screw speed, moisture content, die, and extrusion time can affect protein functionality after extrusion [90]. Studies reported reduced solubility of pea protein isolates during low moisture extrusion. The barrel temperature did not significantly affect the pea protein isolates secondary structure, even though extrusion reduced the ratio of β-sheets to α-helixes and increased the β turns [90]. Structural changes during the extrusion process influence protein solubility, which in turn affects hydrodynamic properties like water absorption, emulsification, and foaming. For instance, increased protein solubility in isolated soy and mung bean proteins leads to higher water absorption capacity. Similarly, extruded faba bean concentrate, with high moisture content during extrusion, exhibits high water-binding capacity due to protein denaturation. Beyond solubility, the balance of hydrophilic-hydrophobic and polar-nonpolar amino acids also serves as a key indicator of protein functionality, including water and fat absorption, as well as surfactant properties [66]. The pea protein isolates subjected to extrusion exhibited enhanced gelation and water holding capacity, making them suitable for high-protein snacks and meat substitutes [14]. The extrusion processing of the protein concentrates of faba beans resulted in improved texture and bite strength in the meat analogues, while also increasing protein digestibility without causing a loss in amino acids or major changes in nutritional value [10,31]. Extrusion cooking causes conformational changes in plant proteins that depend on a number of variables, such as temperature, moisture content, feed rate, screw speed, protein, fat, and carbohydrate content, as well as the inherent characteristics of the plant protein and the addition of polysaccharides and transglutaminase enzymes [66]. Extrusion cooking can alter plant proteins colour, flavour, texture, solubility, digestion, absorption, emulsion properties, water-holding and oil-binding capacities, and so on [130].

#### 7.1.4. Cold Atmospheric Pressure Plasma Treatment

Cold plasma (CP) treatment is a novel non-thermal technology, that can be a potential alternative to traditional thermal processing for microbial inactivation and protein modification [66,128,140]. CP technology is a short-time low-temperature treatment that causes no thermal damage to the food structures. At atmospheric pressure, CP technology generates reactive oxygen species and reactive nitrogen species. These reactive species can trigger lipid peroxidation in microbial cell membranes and oxidise proteins and DNA, leading to microbial inactivation [141]. In addition, these active species can break covalent bonds and initiate chemical reactions [90]. Research shows that cold plasma treatment can modify the secondary and tertiary structures of protein isolate, resulting in a more thermodynamically stable structure. This stability is linked to increased hydrogen bonding within α-helix and β-sheet structures [66]. The unfolding of the protein structure, exposure of active sites, and binding of water micelles to protein molecules enhance water-holding capacity and emulsion stability. Additionally, cold plasma treatment improves functional properties such as solubility and gelling capacity in pea protein [66]. According to Zhang et al. [119] study the efficacy of the atmospheric CP technique as a novel non-thermal technique to improve the gelling properties of pea protein. CP-treated pea protein concentrates (12 wt%) showed good gelling properties at 70–90 °C, while native pea protein concentrate could not form a gel. The treated pea protein concentrate gels exhibited a homogeneous three-dimensional network structure with interconnected macropores. Those prepared at 80 and 90 °C had good mechanical strength and viscoelasticity, as well as high water holding capacity [66]. Observations revealed that CP treatment helps the formation of protein fibrillar aggregates. It also significantly reduced the pea protein concentrate denaturation temperature which led to the unfolding of the protein at a reduced temperature of 80 to 90 °C. Protein surface hydrophobicity was increased, and free sulfhydryl groups were made exposed by the CP treatment. This facilitated the formation of hydrophobic interactions and disulfide bonds, leading to gels with improved mechanical properties. CP is a promising technology to enable wide applications to pulse protein as a gelling ingredient in meat and egg alternatives [66,90]. Pea protein isolates treated with cold plasma demonstrated improved solubility and water-holding capacity, with potential applications in high-moisture food products [22].

#### 7.1.5. Ultrasonic Treatment

Ultrasound, is non-thermal and eco-friendly technology, which uses low frequency and high frequency acoustic waves above 20 kHz to improve the functional properties of plant proteins like soy, pea, and faba bean proteins [90,121,127]. It operates in two modes, these are high frequency low intensity ultrasound (HF-LIU) (100 kHz–1 MHz, power < 1 W/cm^2^) and low frequency high intensity ultrasound (LF-HIU) (16–100 kHz, power 10–1000 W/cm^2^) [83,121]. The mechanism relies on cavitation rapid oscillation between compression and expansion which disrupts macromolecular bonds by creating localised tension and collapse [83]. Ultrasonication has an impact on the biological functions and functional properties of the resultant protein constituents through cavitation-induced heating, dynamic agitation, shear stresses, and turbulence [83,142]. This mechanical action enhances protein functionality, making ultrasound an increasingly valuable tool for protein modification [6,127]. This technique modifies the proteins via localised hydrodynamic shearing and heating of protein molecules in a protein solution. Ultrasound intensity and ultrasound exposure time mainly impact the protein ingredients. Hydrodynamic shearing results in small well-dispersed particles where simultaneous heating could cause thermal degradation. However, a high intensity of the ultrasound wave with low power or prolonged treatment could also induce the aggregation of plant protein extracts suspended in an aqueous solution, leading to larger particle sizes [90,142]. Several studies demonstrate that the power level, frequency combination, and duration of ultrasound have a major impact on the structural integrity of proteins. Prolonged sonication caused the soy protein isolates solubility to first rise before decreasing [127]. Studies have shown that pea protein ultrasonically treated at 20 kHz produced more stable emulsions than untreated samples. Ultrasound increases surface hydrophobicity and decreases particle size, which reduces oil-water interfacial tension and improves foaming characteristics. Additionally studies heighted improved antioxidant properties in pea protein that was extracted by ultrasound [127]. Furthermore, ultrasound works effectively to improve the activity of other legume proteins, including those from peanuts, broad beans, and lentils, as well as cereal and pseudo-cereal proteins. Notwithstanding these developments, the majority of ultrasonic applications in protein modification take place in laboratories. Scaling to pilot or industrial levels is a significant problem [127].

#### 7.1.6. Pulsed Electric Field (PEF)

Pulsed electric field extraction is a novel and non-thermal technology that is being utilised extensively for protein treatment because of its high efficiency, safety, and cleanliness benefits [135]. The PEF technique involves applying high voltage electrical pulses (ranging from 100 to 300 V/cm to 80 kV/cm) to the product between two electrodes in brief bursts (from a few nanoseconds to a few milliseconds) [128,135]. Pulsed electric field modification, which employs high electric field intensities, short pulse widths, and increased pulse frequencies, induces changes in the microenvironment and molecular conformation of protein molecules [6,127]. According to research, pea concentrates treated with a moderate PEF (1.65 kV/cm) showed reduced solubility (from 23.2% to 17.2%) [127]. PEF has the potential to denature and aggregate proteins, most likely by forming S-S bonds and hydrophobic interactions. PEF may also help amino acid side chains cross-link, which would further reduce the solubility of proteins [127]. Solubility is thus affected variably by specific protein types and waveform variations. It has been emphasized how PEF may enhance the flavour characteristic of plant proteins. However, little is known about how PEF affects plant protein digestibility and anti-nutritional factors, suggesting a worthwhile area for further research [127].

#### 7.1.7. Electrospinning

Electrospinning has gained prominence over the last decade as a versatile and scalable technique for fabricating three-dimensional fibrous structures, with applications in tissue regeneration and other fields [143]. This method produces polymer fibres ranging from nanometers to micrometers in diameter, offering a high surface-to-volume ratio, tunable morphology, adjustable surface characteristics, and excellent mechanical properties [144]. These features make electrospinning a promising tool for creating nanofibre and microfibre based materials tailored to specific applications, including mimicking the extracellular matrix to enhance cell function and tissue regeneration [144]. Electrospinning methods extrude polymer solution through a needle by applying an electric potential. Utilizing a powerful electromagnetic force of approximately 20–30 kV, this technology induces ionization in a polymer solution [126,144]. Proteins are mixed with biocompatible natural and synthetic polymers to enable their electrospinnability. The fibre mats that are produced have improved degradability, morphology, mechanical properties, and thermal stability [143]. Solution characteristics including surface tension, viscosity, and conductivity, as well as spinneret operation parameters such temperature, humidity, and spinneret-collector distance, affect the electrospinning process and fibre properties [126].

### 7.2. Chemical Modification

Chemical modification is an unambiguous means to modulate protein functionalities in both academic and industrial communities [110]. Chemical protein modification uses chemicals to make and break bonds amongst proteins and other compounds, and modification by pH alteration. This approach is often used to change the charge or hydrophobicity of proteins. Alternatively, chemical modification can be used to facilitate cross-linking [129,142]. Protein chemical modification involves altering functional groups found in polypeptide chains, such as carboxyl (-COOH), hydroxyl (-OH), sulfhydryl (-SH), and amino (-NH_3_) groups [90,110,127,135]. Two critical features are needed for chemical modification of a protein, that is, an attachable molecule of interest and a practical reaction. The molecule should endow a protein with some specific functions desirable in food applications, whereas the reaction yields the structurally modified protein constructs with functional groups in a high efficiency means [110]. These reactions do not only involve the development of covalent linkages, but also participate in alteration of noncovalent forces including van der Waals forces, electro-static interactions, hydrophobic interactions, and hydrogen bonds, and thereby leading to molecular conformation changes accompanied by intentional functional outcomes [110]. Nonetheless this technique is not widely used in the food industry due to cost, regulations, and clean label trends [90]. These changes affect intramolecular electrostatic interactions, molecular structure, and hydrophobicity, which consequently affect the functional characteristics of proteins, including solubility, foaming capacity, emulsifying properties, gel formation, and thermal stability. Chemical cross-linking, acylation, deamidation, phosphorylation, glycosylation, acid-base modification, and surfactant application are methods for altering food proteins chemically [90,127]. However, chemical means are not as prevalent in the food industry as either physical or biological methods of protein alteration [63].

#### 7.2.1. Acid-Base Treatment/pH Shifting Treatment

The pH shift method is also referred as the isoelectric precipitation method or the acid and alkali solubilization method [71]. Acidic or alkaline treatments can cause changes in the structural and functional properties of proteins since one of the most important elements affecting their structure is the pH of the liquid matrix in which they are dissolved [71,128]. Protein modification using acid-base treatment is common. It uses pH changes that are far from the proteins isoelectric point to improve solubility and other functions [71]. In such conditions, the acquisition of charges on protein surfaces facilitates ion-dipole interactions between charged amino acids and water molecules, hence improving solubility. Additionally, this process causes uniform repulsion across protein surfaces with identical charges, which encourages protein molecules to unfold. Extremely basic pH levels cause proteins to denature and unfold, revealing hydrophobic and sulfhydryl regions in their structure that allow for novel protein interactions. It is simple to create alkaline conditions by adding chemical additions like NaOH, NH_4_OH or urea [128]. According to Zhang et al. [127], high pH levels can change the secondary and tertiary structures of proteins and increase molecular flexibility by rupturing disulfide bonds and intermolecular interactions. According to research, an alkaline pH shift to 12 increased pea protein’s surface activity, resulting in improved emulsion oxidative stability and enhanced adsorption at the emulsion interface. This could be because the protein structure is longer and has more exposed charged groups [127]. Additionally, it was found that treating soy and pea proteins with a pH shift greatly increased their gel strength. This was because the pH shift had the ability to disrupt existing disulfide connections and promote the formation of new ones. Research shows that proteins in extremely acidic or alkaline environments become more sensitive to temperature, even though pH shift operations are usually carried out at ambient temperature [127]. Although acid-base modification has advantages, it is important to be aware of its possible disadvantages, such as the possibility of unwanted chemical residues that could jeopardise the safety and quality of changed plant proteins. Furthermore, nutritional content and health benefits may be diminished if essential components are lost. In order to satisfy customer demands and legal regulations, producers and researchers are always working to improve these procedures [127].

#### 7.2.2. Glycation

Glycation is a common food-grade reaction which is widely used to improve protein functionalities because it is safe, and it usually does not require exogenous chemicals [142]. Glycosylation refers to the attachment of carbohydrate moieties to amino acid residues, commonly lysine or the N-terminus of proteins, often occurring through the Maillard reaction [90,110,127]. This reaction, a thermally driven process between amino groups and carboxyl-containing moieties, results in structural and functional alterations in proteins. Glycation, a subset of glycosylation, involves a nonenzymatic reaction between the reducing sugars and the amino groups of proteins, typically targeting lysine residues [110]. These modifications can enhance protein functionalities, including improved solubility, emulsification, and thermal stability [110]. During the Maillard reaction, active amino acid residues such as the ε-amino group of lysine and the guanidino group of arginine act as primary reaction sites, leading to changes in protein charge and conformation that influence their overall properties and applications [110]. Both dry-heat and moist-heat techniques can be used to carry out this procedure. Dry-heat glycosylation involves dehydrating a protein and polysaccharide mixture in an aqueous solution, then heating it under particular conditions. The process stops when the mixture reaches room temperature. This approach has a longer time limit even if it produces a higher grafting rate. The moist-heat method, which offers a faster reaction time but lower grafting efficiency and more by-products, requires heating a pre-mixed solution of proteins and polysaccharides at a predetermined temperature [127].

Glycosylated foods must enhance functionality while minimising adverse effects on flavour and colour, necessitating precise control of the Maillard reaction to prevent undesirable sensory changes [127]. Among chemical modifications, non-enzymatic glycation via the Maillard reaction is a key pathway for altering protein functionality and developing flavour. This reaction occurs between carbonyl groups and amino groups, producing the characteristic aromas and flavours enjoyed by consumers. However, it also poses the risk of generating off-flavours, highlighting the importance of controlled processing conditions to balance functional improvements and sensory quality [110]. For example, researches have shown that the dry-heat grafting reaction improves the emulsifying properties of soy proteins with citrus and apple pectin, and that treating pea protein isolates with gum arabic at 60 °C improved their solubility and emulsification ability. Glycated yellow pea proteins solubility, emulsification, and antioxidant activity were all enhanced as a result. According to Zha et al. [110], the grafted polysaccharides enhanced steric hindrance rather than electrostatic repulsion is the primary source of the pea proteins improved functioning. Despite its efficiency, the glycosylation procedure can produce byproducts such as lanthionine, acrylamide, furan, pyruvaldehyde, and 5-hydroxymethylfurfural (HMF) that jeopardise the end products safety [127]. While glycation improves the functional properties of pulse proteins, it can also lead to the formation of advanced glycation end products, which are associated with potential health risks [110]. It was really mentioned that in addition to lysine, other proteinogenic amino acids including arginine, tryptophan, and methionine may also be broken down during the Maillard reaction. To compensate for the lost nutritional value, intact proteins or the appropriate essential amino acids could be added. Remarkably, heating proteins can also increase their nutritional value if, for instance, denaturation improves digestibility or enzyme inhibitors are inactivated. Certain dietary foods, such as acrylamide and heterocyclic aromatic amines, have been found to contain Maillard compounds that may cause cancer [110]. These harmful substances may interfere with the proper structures and activities of proteins, compromising the nutritional value and safety of food. They might also be linked to a higher chance of developing cancer. Controlling the glycosylation processes variables, including temperature, pH, and reactant concentrations, is therefore essential. Comprehensive safety evaluations of the end products must also be carried out. These results emphasise that employing glycosylation requires careful thought [127].

#### 7.2.3. Acylation

Acylation involves the transfer of an acyl group to the amino or hydroxyl group of amino acid residues. There can be different forms of protein acylation like succinylation, acetylation, maleylation, or palmitoylation, depending on the acylating agent and the amino/hydroxyl group [90,128]. Acetylation shifts the isoelectric point of proteins to a more acidic pH, reflecting an increase in electronegativity due to the neutralisation of protonated amino groups and the unmasking of carboxylate groups [110,122]. This process reduces the electrostatic attractions of the proteins, initiating the partial unfolding of the polypeptide chains and resulting in a more expanded structural state [110]. These structural changes from acylation impact protein functionality, notably enhancing aqueous solubility in the pH range between the isoelectric point of the native protein and alkaline pH levels [110]. Using succinic anhydride acylation, researchers were able to increase the solubility and emulsion properties of mung bean protein isolates. Similar improved functionality has been obtained with the pea protein isolate by succinic anhydride acylation. Modification by acylation reduces the net surface charge and the spatial structure of the protein, increases the balance of the aromatic aliphatic residues, and unfolds the polypeptide chain, leading to increased protein flexibility [90]. It was clearly documented that faba bean acetylation resulted in partial dissociation of 11 S globulin into 3 S subunits and a significant decline in α-helical structure. Notably, both conformation and association–dissociation state of proteins seem to be closely associated with the extent of acetylation [110].

#### 7.2.4. Succinylation

Succinylation is a protein modification process where succinic anhydride reacts with amino groups (-NH_2_) on proteins, particularly lysine residues, forming succinylated proteins. This modification introduces succinyl groups (-COCH_2_COOH) onto the protein, altering its physicochemical properties and functionalities [83] and increasing its net negative charge due to the addition of carboxyl groups [110]. Unlike acetylation, succinylation creates a more significant negative charge. The process involves a nucleophilic attack by the protein’s amino group on succinic anhydride, forming an unstable intermediate that rearranges into a stable succinylated protein and succinic acid [83]. This results in the formation of an unstable intermediate, which then undergoes intramolecular rearrangement to yield a stable succinylated protein and a molecule of succinic acid [83]. Furthermore, the electrostatic repulsion that develops between adjacent carboxyl groups causes the protein to dissociate gradually and progress into a more widespread unfolding phase [110]. According to reports, during consecutive succinylation, 11 S globulins of the pea protein eventually disintegrate into 3 S subunits via a trimeric 7 S intermediate. When 7 S vicilin from kidney beans was succinylated, it was observed that the tertiary conformation changed significantly, and the β-sheet progressively transformed into an α-helix or random coil [110]. Research indicates that succinylation modification of chickpea protein enhances its functional properties, including water-holding capacity, oil-holding capacity, solubility, and emulsifying capacity. These improvements result from structural and surface property changes caused by succinylation [83].

#### 7.2.5. Phosphorylation

One method of chemically modifying plant-based proteins that preserves their nutritional bioavailability is phosphorylation. The process of adding phosphate groups to a proteins basic sequence, known as phosphorylation, can significantly control how that protein functions [128]. Phosphorylation is crucial chemical processes for altering food proteins. An enzyme known as protein kinase catalyses the covalent bonding of the phosphate moiety to a particular amino acid residue of proteins, changing the proteins shape and altering its stability and activity. Additionally, phosphorylation can alter interactions with other proteins by permitting other proteins to bind via the phosphorylated regions [128]. Protein functionality, including emulsifying ability, solubility, and stability, can be greatly improved by this method [127]. Phosphorylation is the covalent attachment of phosphoryl groups (PO_3_^−^) to specific reactive amino acid residues (-NH, -OH, or -SH) in protein molecules [90,110,127,128]. This modification increases the hydrophilicity of the protein by deprotonating the molecule and introducing negative surface charges, leading to enhanced solubility [90,127]. The degree of phosphorylation is influenced by factors such as the type of protein, the phosphorylating agent (e.g., sodium tripolyphosphate, sodium trimetaphosphate, or POCl_3_), and the reaction conditions [90]. According to Liu et al. [145], phosphorylation markedly enhanced the solubility, emulsifying, foaming, and oil absorption ability of the pea protein isolate [110,127]. However, because of their harsh reaction conditions and nonspecific chemical reagents, chemically phosphorylated proteins are less desirable for food applications and have not been easily embraced by health-conscious customers. Furthermore, the degree of phosphorylation increased the phosphorylated soy protein isolates apparent molecular weight and thiol content, which had a detrimental effect on gastrointestinal digestibility, especially stomach digestibility [110,127]. Therefore, it is important to carefully analyse the possible toxicity of residues added to legume proteins in order to use phosphorylated proteins as food ingredients in a safe manner. To assess the toxicity and physiological roles of phosphorylated proteins, mammalian experiments are necessary [127]. In work [145] it was reported that pH 12, 70 °C and 7.0% (*w*/*v*) sodium tripolyphosphate as the optimal condition for pea protein isolate phosphorylation. Under these conditions, the solubility, emulsifying properties, foaming properties, and oil absorption capacity of the modified pea protein isolate were significantly improved. They also observed modified pea protein isolate having a smooth and uniform lamellar structure, where the content of α-helix and β-sheet structure increased, but the content of β-corner and random coil structure decreased [90]. More recently, another feasible mean has been portrayed, namely, phosphorylation-reactive hydroxylated amino acid residues by dry-heating the homogeneous mixture of proteins and orthophosphate or pyrophosphate [110]. Accumulated evidences have indicated that phosphorylation leads to changes in the architecture of proteins, legitimately ascribing to the electrostatic repulsion from incorporated phosphate groups. Accordingly, protein functionalities such as heat stability, emulsification, foaming, and gelling properties were improved with the exception of a slight decrease in water solubility [110]. Future study should focused on elucidating the nature of phosphate linkages and phosphorylation sites in pulse proteins. Phosphorus content that can be introduced in proteins is low, and <1 g/100 g proteins can be achieved by dry-heating (85 °C and different pHs) [110].

#### 7.2.6. Cross-Linking

Crosslinking of proteins through transglutaminases and other oxidative enzymes can improve some of the functional properties. Transglutaminases enzyme catalyses the acyl transfer reactions and crosslinking between glutamine (acyl donor) and lysine (acyl acceptor) [90]. One study showed with transglutaminas crosslinking, the electrostatic stability of faba bean protein isolates improved, but the solubility of the protein decreased. Also, the foaming properties of faba bean protein isolate decreased with transglutaminases enzyme treatment, due to increased dynamic surface tensions and impaired adsorption at the air-water interface [90]. Crosslinking increased the particle size and polydispersity, leading to the formation of a gel-like structure. The addition of enzymes improved the physical stability of the emulsions. Transglutaminases crosslinked chickpea stabilised emulsions showed decreased digestibility. Transglutaminases treatment also can significantly increase the gel strength of protein isolates [90].

#### 7.2.7. Esterification

One important chemical technique for altering proteins in food is esterification. When free carboxyl groups are inhibited by esterification with different alcohols, the basicity and net positive charge of the modified proteins are raised. This technique can significantly enhance protein functionality, including emulsifying capacity, solubility, and stability. For instance, two pulse proteins (chicken and broad bean) became more soluble in the pH range of 2 to 5 after esterification [127]. Furthermore, these esterified pulse proteins outperformed the original proteins in terms of emulsification and foaming in the acidic pH range of 2 to 6. According to studies, one practical method to improve these characteristics may be to modify the isoelectric point of the protein by esterification. Under weakly acidic (pH 5) conditions, all samples of esterified protein nanoemulsion showed small mean particle sizes, significant stability, and antibacterial inhibitory activity [127].

#### 7.2.8. Deamidation

Deamidation occurs when proteins react with acid or alkaline at increased temperature and results in the conversion of δ or γ amide groups to carboxylic groups with the loss of an ammonia molecule. Usually mild alkaline or acid deamination is preferred due to low protein denaturation and peptide hydrolysis [90]. Deamidation is the process of removing amide groups from asparagine or glutamine residues in proteins, resulting in the formation of negatively charged carboxyl groups [90,128]. This modification increases the net negative charge on the protein, which lowers its isoelectric point and enhances solubility, particularly in acidic environments. The increased negative charge also reduces protein aggregation and improves functional properties such as emulsifying capacity by increasing electrostatic repulsion between protein molecules [10,90]. Enzymatic deamidation, catalysed by glutaminase, specifically targets glutamine and asparagine residues, leading to improved solubility and functionality without extensive protein hydrolysis [90,128]. Furthermore, derivatising non-essential amino acid residues like aspartate and glutamate can enhance the nutritional quality of proteins compared to modifying essential residues like -NH_2_ of lysine or -OH of threonine [110]. Amidation or esterification of the β- and γ-carboxyl groups of aspartate and glutamate can convert these negatively charged amino acids into positively charged derivatives, potentially transforming acidic proteins into basic ones and offering a means of further modulating protein functionality [10,110]. This process increases the net negative charge on the protein molecule, leading to improved solubility, especially in acidic environments. This charge alteration also reduces protein aggregation and improves emulsifying capacity by increasing electrostatic repulsion between protein molecules [10]. Enzymatic deamidation is preferable especially due to mild reaction conditions. Studies shown the feasibility of improving the properties of pea protein isolate by deamidation with glutaminase. The structure profile revealed that the glutaminase treatment does not change the basic protein subunit composition. Protein unfolding and conformational reorganisation with increased β-turn structure and reduced β-sheets and antiparallel β-sheets, led to more flexible, small size, more hydrophobic groups exposed proteins. This modification also improved the sensory properties of the pea protein isolate [90]. Proteins from wheat, corn, barley, and soy have been chemically deamidated in the majority of previous research, but not those from pulses. According to these earlier findings, a controlled deamidation level of less than 45% from cereal proteins and 72% from soy proteins can result in good functionality [90]. Glutaminase deamidation of pea protein isolates allows the protein to unfold and undergo structural reorganisation without altering the fundamental protein composition. In comparison to the untreated, the deamidation results in pea proteins that are more flexible, soluble, homogeneous, and dispersible while also having less lumpiness, grittiness, and beany flavour. Therefore, a possible method of improving the applicability of pea proteins is glutaminase treatment [14].

### 7.3. Biological Modification

Biological modification techniques, involving the use of microbes or enzymes, provide a sustainable and effective way to improve the functional properties of pulse proteins, hence overcoming their functional limits [128]. Enzymatic hydrolysis, microbial fermentation, and germination that can selectively modify the structure and function of proteins are usually involved in this process [63,69,128,142]. The biological modification technique is environmentally friendly and less energy-consuming without production of toxic by-products [128]. In addition to modulating protein functionality, these approaches have the ability to improve their nutritional quality, including digestibility, bioavailability, antioxidant and antimicrobial properties, as well as can decrease the amount of anti-nutritional factors [79,128,146]. Furthermore, biological modification can enhance the flavour and texture of pulse proteins, making them more suitable for a wider range of food applications [146].

#### 7.3.1. Fermentation

One of the oldest and most economical biological processes for modifying plant-based proteins is fermentation [128]. This technique is particularly beneficial for plant-based proteins, such as those derived from legumes, which often contain antinutritional factors [147]. Fermentation can improve the digestibility of plant proteins by breaking down complex compounds and reducing antinutritional factors like phytic acid, trypsin inhibitors, α-galactosides, lectins and chymotrypsin inhibitors. This process increases the bioavailability of essential amino acids and minerals (calcium, iron, magnesium, and zinc), making nutrients more accessible for absorption [147,148,149]. This reduction enhances the overall nutritional profile of fermented foods [147,149]. Various starter cultures, including lactic acid bacteria, yeast, mold, and *Bacillus strains* of which lactic acid bacteria are the most prevalent, have been used to ferment plant proteins [128]. Fermentation is also considered an effective biological method for improving functional properties, increasing bioavailability, producing bioactive peptides, and expanding the range of plant protein utilization. Lactic acid bacteria (LAB) (e.g., *Lactobacillus*, *Staphylococcus*, *Enterococcus*, *Micrococcus*, etc.) and certain fungi (e.g., *Candida* spp.), are mainly used to ferment plant-based proteins [69]. Fermentation is also method that is used to optimise nutritional properties and functional properties and to reduce off-flavors [146,150]. During fermentation, microorganisms produce enzymes that create proteolytic cleavages in protein and breakdown into lower molecular weight amino acids and peptides [90]. As studied shown the impact of *Lactobacillus plantarum* fermentation on the nutritional properties of pea protein concentrates. They observed fermentation influencing the anti-nutrient compounds leading to a decrease in protein quality. The phenol and tannin content increased with fermentation and the inhibitory activity of digestive enzymes decreased. Although protein digestibility increased, alteration of sulfur amino acid content resulted in an overall reduction in protein quality [90]. Additionally, studies have demonstrated that fermenting lupin and pea protein isolates with *Lactobacillus* species increases protein solubility, which is advantageous for various food applications [149]. In contrast, impact of fermentation on protein content can vary depending on the substrate and microbial strains used. Some studies have observed a decrease in protein extraction yield due to protein hydrolysis during fermentation, which may lead to inconsistent results [151]. Overall, trypsin and chymotrypsin inhibitor activities decreased over the duration of fermentation, due to the production of hydrolytic enzymes. These data suggest the use of alternative bacterium for fermentation which can increase the digestibility by reducing anti-nutrients and which metabolises sulphur amino acids to a lesser extent [90]. During fermentation, the amounts of indigestible α-galactosides and phytic acid were reduced by 90% and 17%, respectively in pea protein isolates. In another study, air-classified pea protein concentrates (52%) subjected to fermentation for 120 h using six different fungal organisms (*Aspergillus niger*, *Aspergillus oryzae*, *Aureobasidium pullulans*, *Neurospora crassa*, *Rhizopus microspores* var. *oligosporus*, *Trichoderma reesei*) have been shown to increase total phenolic content, protein content, protein solubility, saponin content, and fibre fractions [114]. The water-holding capacity of the freeze-dried sourdough was increased compared to the original protein material. The foam capacity was reduced, due to partial hydrolysis of protein during fermentation. Shi and coworkers [152] reported lower water-holding capacity, higher oil-holding capacity, increased foaming properties, changes in colour and density and no significant difference in emulsion properties in pea protein isolates with lactic acid fermentation [90]. Fermentation can be used as a lever to reduce off-flavours and improve sensory perception. According to Youssef et al. [153] study a starter co-culture of LAB with *Kluyveromyces lactis*, *Kluyveromyces marxianus*, or *Torulaspora delbrueckii* on a 4% pea protein solution. The sensory evaluation showed a significant reduction in the leguminousattributes of pea proteins and the generation of new descriptors in the presence of yeast. Compared to the non-fermented matrix, fermentations with LAB or LAB and yeasts led to the degradation of many off-flavour compounds, and the presence of yeasts triggered the generation of favourable esters. During storage, lupin protein extracts are prone to the development of beany off-flavours which confines its application in foods [90]. As studies have shown, fermentation of lupin protein extract using several LAB indicated a significantly modified aroma profile where the off-flavours were reduced and/or masked by newly formed compounds [90]. These results indicate that the final nutritional, sensory, and functional product characteristics are greatly affected by fermentation conditions such as microbial culture, fermentation time, and the type of protein ingredient [90]. Lactic acid fermentation has been applied to reduce the beany odours of pea protein concentrates. However, the effectiveness of this process depends on the concentration of pea protein concentrate (0 to 40% addition) and the type of starter culture used (10 types studied). Although green/honey flavours can be reduced, undesirable characteristics such as astringency and bitterness can increase during fermentation [14]. Fermentation with Lactobacillus plantarum induces protein hydrolysis, generating novel flavours, reducing antinutritional factors, and increasing bioactive peptides. This method also allows tailoring of the functional properties of fermented proteins based on pH and fermentation duration [14].

Similarly, the lactic acid fermentation of faba bean flour enhances its nutritional profile by hydrolyzing proteins and modifying their functional properties. Fermentation improves protein digestibility, lowers the glycemic index, and eliminates antinutritional factors such as trypsin inhibitors, phytic acid, saponins, and galactosides. Studies indicate that fermentation significantly boosts in vitro protein digestibility while reducing trypsin inhibitor activity [154]. It also reduces antinutritional compounds like vicine, convicine, and α-galactosides (e.g., raffinose, stachyose, and verbascose), improves the concentrations of beneficial amino acids (cysteine and methionine), and enhances functional properties such as solubility, foaming capacity, and stability [31]. However, fermentation of pea proteins may lead to reduced solubility and foaming properties, though emulsification capabilities remain unaffected [75]. Fermentation is widely used to extend the shelf-life of foods while enhancing their aroma, flavour, texture, and nutritional quality. It can be applied at both the ingredient level (e.g., protein isolates) and the product level (e.g., plant-based milk). Lactic acid bacteria fermentation is the most studied process, while fungal and yeast fermentations are less explored. LAB strains like *Pediococcus pentosaceus*, *Leuconostoc kimchi*, *Weissella cibaria*, and *Weissella confusa* have been isolated from faba beans, while *Leuconostoc plantarum*, native to legumes and grains, is the most studied strain for fermenting faba beans and peas [31]. In addition, fermentation has been used to successfully remove off-flavors in soy and similar effects have been observed in pea protein and pea-based milk analogues. LAB can alter protein composition and structure through enzymatic activities like proteolysis, releasing bioactive peptides that offer health benefits such as ACE-inhibitory, antioxidant, antidiabetic, and immunomodulatory activities. Overall, fermentation enhances the physicochemical, nutritional, functional, and sensory properties of legume proteins [75]. Furthermore, fermentation can improve the functional properties of proteins, such as solubility, water/oil holding capacity, emulsification and texture-forming properties [10,147]. Studies have demonstrated that fermenting lupin and pea protein isolates with *Lactobacillus* species increases protein solubility, which is advantageous for various food applications [149]. In contrast, impact of fermentation on protein content can vary depending on the substrate and microbial strains used. Some studies have observed a decrease in protein extraction yield due to protein hydrolysis during fermentation, which may lead to inconsistent results [151]. Additionally, the acidification process during fermentation can induce protein aggregation and denaturation, potentially affecting the texture and functionality of the final product. Moreover, microbial production of organic acids during fermentation promotes mineral absorption by preventing the formation of insoluble mineral-phytate complexes [10]. For example, lactic acid produced by lactic acid bacteria can decrease the surface charge of proteins like casein, leading to aggregation [10,155].

#### 7.3.2. Enzymatic Modification

Enzymatic modification involves the use of enzymes to alter polypeptide chains and amino acid residues in proteins, enhancing their structural and functional properties [127]. This method has been widely applied to modify plant proteins, primarily by breaking them down into desirable peptides, thus tailoring their functional characteristics [110,121,150]. Proteins can be modified through the controlled application of proteolytic and non-proteolytic enzymes [97]. Enzymatic modification allows one to achieve desired characteristics by inducing dissociation, association, or structural changes in proteins [9,90]. Proteolytic enzymes like pepsin and trypsin are used to hydrolyze peptide linkages in the protein primary amino acid sequence. Non-proteolytic enzymes like transglutaminases are used to associate proteins through crosslinking [90,97]. Enzymes such as glutaminase can be used to deamidate proteins as change amide groups to carboxylic groups [90]. Enzymatic hydrolysis is a promising method for modifying the structure of plant proteins to enhance their techno-functional and sensory properties, and to reduce antinutritional factors [63,79,93]. This process increases surface hydrophobicity and exposes ionizable groups, improving interactions with water and oils in food systems. Enzymatic hydrolysis has been reported to improve pulse protein functionality due to the smaller size of protein hydrolysates [90]. This method reduces the molecular weight of proteins, leading to improved solubility. However, as the degree of hydrolysis increases, interfacial tension and surface charge decrease, which can negatively impact the emulsion properties [63]. This drawback is attributed to the inability of short-chain peptides to flexibly form stable interfacial films around oil droplets [63,90]. Despite this, enzymatic hydrolysis improves solubility, as well as foaming and emulsifying capacities, across various pH levels [31]. For example, studies show that the faba bean protein exhibits improved emulsifying properties after enzymatic treatment, making it suitable for replacing egg yolk in low-fat mayonnaise. Similarly, enzymatic treatment of pea flour with acid protease improves solubility and oil absorption by exposing ionizable amino and carboxyl groups. According to research, tyrosinase and transglutaminase are used to hydrolyze soy protein to increase its viscosity and hydrophobicity and produce a protein-based film with exceptional water-holding capacity and tensile strength [5]. Enzymatic hydrolysis has been used to enhance specific functional properties of plant proteins, such as the foaming properties of soybean protein, the surface hydrophobicity of sunflower protein and the emulsifying properties of rapeseed protein [121]. It is preferred over other modification methods due to its mild process conditions, including moderate temperature and pH. Enzymatic hydrolysis is extensively employed to tailor protein functionality for specific applications, often improving solubility in plant proteins like pea and faba bean proteins. However, its effects on other functional properties, such as emulsifying and foaming capacities, depend on the protein type and degree of hydrolysis [121,142]. While enzymatic hydrolysis generally improves foaming capacity and emulsifying activity, the resulting foams and emulsions are often less stable [121]. Controlled hydrolysis, by decreasing molecular weight and exposing hydrophobic regions, can enhance the ability of proteins to form and stabilise emulsions where improvement is needed [9]. Proteolysis of faba bean proteins at neutral pH with enzymes like pepsin, trypsin, flavourzyme, alcalase, or neutrase significantly improved protein solubility, foaming capacity, and oil-holding ability [154]. The effects of enzymatic hydrolysis on foaming properties vary considerably depending on factors such as enzyme type, substrate, degree of hydrolysis, and pH [69]. Hydrolysis is particularly effective for improving foaming capacity near the proteins isoelectric point, where increased solubility plays a key role [9]. For instance, studies have shown that the foaming capacity of faba bean proteins improved to varying degrees at pH 5 with enzymes like pepsin, trypsin, flavourzyme, or neutrase, while at pH 7, the impact on foaming capacity depended on the specific protease and hydrolysis duration [9]. Enzymatic hydrolysis is particularly effective for improving the solubility and functionality of proteins with low initial performance, such as pea protein. By reducing molecular size and exposing hydrophilic groups, hydrolysis enhances solubility, oil absorption, and emulsification, making the hydrolyzed proteins more versatile for use in various food products. Proteolytic enzymes like papain and α-chymotrypsin are commonly used to produce desirable hydrolysates without degrading amino acids [9]. The advantages of enzymatic hydrolysis include its specificity, controlled processing, and mild conditions, which help preserve the nutritional quality of amino acids. This makes it a favourable alternative to chemical hydrolysis. Enhanced protein solubility achieved through enzymatic hydrolysis is particularly valuable for food applications [9]. Enzymatic hydrolysis is a promising technique for producing bioactive peptides from plant proteins, offering various biological properties such as antihypertensive, antibacterial, and antioxidant effects [5,97]. For instance, peptides derived from hydrolyzed pea protein have shown potential as treatments for renal illnesses, demonstrating the ability to inhibit Escherichia coli and Staphylococcus aureus while functioning as trypsin and chymotrypsin inhibitors [5]. The increasing recognition of plant peptides and proteins as valuable ingredients in innovative food products underscores their growing importance [5]. Despite its advantages, enzymatic hydrolysis poses challenges, including the formation of unwanted bitter flavoures and high enzyme costs, which could limit its broader adoption in food applications. Additionally, while enzymatic hydrolysis is often preferred over chemical hydrolysis due to its milder conditions, easier process control, and preservation of nutritional quality, further research is needed to fully understand its impact on the quality of food products and formulations [5]. Enzymatic hydrolysis also offers improved digestibility and antioxidant activity; for example, lentil protein hydrolysis has been shown to increase in vitro protein digestibility. As pulse protein ingredients gain wider use in the food industry, understanding enzymatic tools and strategies to enhance their functionality will be critical to expanding their applications. This review focuses on the current knowledge of enzymatic hydrolysis’s effects on key techno-functional properties of high-protein pulse ingredients and its potential to improve these characteristics [9,97]. Enzymatic hydrolysis can significantly influence sensory properties, such as bitterness and astringency, due to the release of low molecular weight peptides. The efficiency of hydrolysis depends on various factors, including the type of enzyme, hydrolysis conditions (time, temperature, and pH), and the degree of hydrolysis [93]. This process improves protein solubility, enhances emulsifying and foaming capacities, and provides a tailored approach to modifying proteins for diverse food applications [9]. The effects of enzymatic hydrolysis are highly dependent on the enzyme type and treatment conditions. For example, the functional properties of enzymatic hydrolysates from pea protein isolate vary significantly based on the degree of hydrolysis and the enzyme used [53]. In conclusion, this method is particularly useful in overcoming the challenges presented by pulse proteins, which frequently have poor solubility, antinutritional components, and undesirable sensory properties. Furthermore, enzymatic treatment also plays an important role in reducing bitter flavours and ameliorating allergies to plant proteins [69]. Given the increasing demand for pulse protein components in the food industry, it will be crucial to understand enzymatic hydrolysis in order to maximize their functional and sensory attributes for a range of applications [93].

#### 7.3.3. Germination (Sprouting)

Natural and spontaneous, seed germination is a biological process that involves a number of physiological and biochemical processes. During germination, endogenous enzymes mobilize and modify stored proteins, supplying vital nutrients for plant growth [156]. According to He at al. [156], germination may cause modifications to the secondary structure of proteins, which would include a decrease in β-turns and irregular coils and an increase in α-helices and β-sheets. Germination is used to improve the nutritional and tech-functional properties of plant proteins [146]. During germination, higher molecular weight proteins are broken down as proteases and amylases are activated [69,156]. For example, after three days of germination, the storage proteins of hemp seeds were significantly broken down, changing their surface charge and improving their water-holding capacity, foaming, emulsifying, and gelating properties [156]. Due to the increased in vitro digestibility of chickpea and sesame proteins, germination has also been shown to boost antioxidant activity, B-vitamins, and the digestibility of legume proteins in addition to its techno-functional properties [50,156,157]. The most pronounced changes have been found in the water absorption, emulsifying, and foaming properties of flours made from germinated pulses [50]. Germination has been considered as a promising approach for generating novel bioactive peptides. During germination, endogenous proteases hydrolyze storage proteins, changing their structure [156].

#### 7.3.4. 3D Printing

Three-dimensional/3D printing (3DP) is a novel technology that has vast potential in creating instrumental change in food and agricultural sector [126]. Three-dimensional (3D) printing technique involves, pushing the material through a nozzle and depositing it layer-by-layer to produce the required structure [158]. The difficulty lies in developing a material that functions as a solid at rest to support the printed structure while flowing when stress is applied to pass through the nozzle. According to Sridharan at al. [158], materials possessing these rheological characteristics are referred to as plastic or yield stress materials. 3D printing can be used to create scaffolding for tissue cartilages and specialised functional foods, among other things. Currently food printing is mostly performed using carbohydrate polymer materials, with added cross-linking agents and other natural fillers such as vegetables or chocolates. 3D printing is an innovative method that enables the development of food products with personalised forms and nutritional characteristics, in addition to producing conventional foods with appealing designs [158]. Materials for 3D printing must be easily extrudable through the nozzle, shear-thinning, and maintain the deposited shape [31,158]. By utilizing 3D printing technology, plant proteins can be given desired fibrous and complex muscle-like shapes [126]. It is possible to create products and structures based on animal proteins that are incredibly flexible in terms of their geometric patterns, flavours, sensations, and nutrients. Reduced particle size and flavour dilution are necessary for 3D printed product production, which lowers the value of premium meat products. As an alternative, this method might be a better way to use tough, low-value meat trimmings and cuts [126]. The emulsifying and water-and fat-binding properties of pea proteins make them a good ingredient for 3D printing [31]. In faba bean 3D-printed products, fibre is an important ingredient because of its high water-binding capacity, which improves printability and shape stability [31]. This technology is highly sustainable and energy efficient because it uses raw materials efficiently with little to no waste, makes it easier to control the product’s composition and nutrition, uses unique and exotic plant proteins, saves time and labour, and manages the supply chain with transportation by moving the production facility close to the area of consumption [126]. Table 6 summarises potential pulse protein modification methods along with their benefits, drawbacks, and impacts on techno-functional properties.

## 8. Limitations for Human Consumption

Field bean and field pea are excellent sources of plant-based proteins, offering high nutritional value and health benefits. However, there are several limitations and challenges associated with their consumption. These issues primarily stem from their composition, particularly the presence of anti-nutritional factors, low digestibility, and allergenic potential [30,40].

### 8.1. Antinutritional Factors

Antinutritional factors have a wide range of biological functions; their presence in pulses is part of an adaptation mechanism to protect them from adverse environmental conditions as well as providing a defence mechanism against insects. They can negatively affect the nutritional quality and bioavailability of nutrients but also exhibit health-benefiting properties. The antinutritional content of pulses depends on a range of factors such as genotype, seed maturity, seed tissue, environment, growing region, and seasonal variation [114,159]. The common anti-nutritional factors present in pulses are enzyme inhibitors, such as trypsin inhibitor, chymotrypsin inhibitor and α-amylase inhibitor, lectins, tannins, phytic acid, oxalates, phenolic compounds, saponins and oligosaccharides (flatulence factors) [11,12,14,24,120,159,160]. Distribution of these ANFs in different components of pulses also varies. Phytic acid, oligosaccharides and enzyme inhibitors are mainly concentrated in the cotyledons of pulses. Tannins and phenolic compounds are mainly located in the seed coat of pulses [160]. ANFs compounds have both positive and negative effects, depending primarily on their concentration [159]. Anti-nutritional compounds like trypsin inhibitors, lipoxygenase, phytates, lectins, and α-galactosides are often found in the soluble albumin fraction after separation from globulins in legumes [75] (Table 3). These compounds can interfere with nutrient availability and digestion, potentially causing health issues. For example, trypsin inhibitors inactivate the digestive enzyme trypsin, reducing protein digestibility, amino acid absorption, and mineral bioavailability. Similarly, α-galactosides, part of the raffinose family of oligosaccharides (RFO), are fermented by gut bacteria, leading to flatulence and digestive discomfort [75]. Phytic acid binds to proteins, enzymes, and minerals, reducing their solubility and absorption, which can lead to nutrient deficiencies, such as iron deficiency anaemia, in populations heavily reliant on pulses [75]. In addition, phytates and polyphenols inhibit the absorption of minerals like calcium, iron, and zinc [24]. Legume cultivars vary in their levels of anti-nutrients, which makes it important to screen varieties carefully to identify those that are best suited for food or feed applications [24]. While pulses are nutritionally beneficial, their digestibility and sensory properties can be negatively affected by anti-nutrients [25]. Advances in concentration and isolation techniques have helped improve the nutritional and functional properties of pulse-derived ingredients for food products [15].

The limited use of faba beans is largely due to the presence of anti-nutritional factors and their adverse effects on human and animal health. These compounds reduce nutrient absorption and digestibility and can cause specific health issues [2,27,31,47]. The most significant ANFs in faba beans include vicine, convicine, tannins, phenolics, phytic acid, saponins, oligosaccharides, trypsin and protease inhibitors, and lectins (favin), which restrict their use as a protein source [27,30,47,63]. Vicine and convicine, natural pyrimidine glycosides, are particularly problematic as they can induce favism (hemolytic anaemia) in individuals with glucose-6-phosphate dehydrogenase (G6PD) deficiency [2,16,21,27,31,87]. Faba beans are also rich in galacto-oligosaccharides (GOS). GOS are included in the Fermentable Oligo-, Di-and Monosaccharides And Polyols (FODMAP) family. FODMAP are poorly digestible and can lead to gas and fluid accumulation in the gastrointestinal tract. This property makes faba beans less suitable for individuals with irritable bowel syndrome (IBS) unless they adhere to a low-FODMAP diet [2,27,31,47,87]. Different faba bean varieties exhibit verbascose, raffinose, and stachyose concentrations of raffinose-family oligosaccharides. Due to anaerobic fermentation by digestive tract microbes, RFO have been linked to pain and flatulence, among other negative health effects. In the meantime, it has been demonstrated that low RFO levels are good for human health. RFO’s prebiotic qualities, which boost the number of bifidobacteria in the gut microbiota, facilitate mineral absorption, strengthen the immune system, and prevent colon cancer, are examples of its health benefits [8]. Similar to faba beans, there are a number of obstacles to using pea protein in the food industry, such as its complicated globular shape, limited solubility, unfavourable flavour components and anti-nutritional elements that hinder digestibility [21,75]. Peas popularity as a common source of protein is hampered by anti-nutritional factors and high levels of off-flavour components that reduce protein quality [161]. Despite not being on the list of the main allergens, there is evidence in the literature that cooking pea proteins can make them more allergenic (Pis s 1, Pis s 2, and Pis s 3; [9,93]. Nevertheless, interestingly, recently many of these ANFs have demonstrated several health benefits and have the potential to become active ingredients for the food industry [159,160]. Various studies have indicated that frequent consumption of pulses such as beans, lentils, field peas and chickpea peas can reduce the risk of cardiovascular disease, and diabetes, and may have protective effects against numerous types of cancers such as breast cancer, colon and rectal cancer. Specific components responsible for these beneficial effects are still under investigation. Studies also suggest that some components in pulses traditionally considered to be antinutritional may have positive health benefits. For example, phytic acid has antioxidant, anticancer, DNA protective effects, chromatin remodelling, endocytosis and hormone signalling; lectins may play a key role in preventing certain cancers, have anti-immunomodulatory activity and may be used for controlling obesity; some protease inhibitors have been shown to have anti-inflammatory properties; phenolic compounds including phenolic acids and flavonoids exhibit antioxidant properties; saponins are known to possess an array of beneficial effects such as lowering plasma cholesterol levels in humans and possessing anticancer properties, as well as being crucial in reducing the risk of various chronic diseases; and resistant starch, and indigestible oligosaccharides may exert prebiotic effects. Oligosaccharides display physiological benefits like bowel function and immune health, reduce chances of coronary heart diseases, increase lactobacilli and bifidobacteria population and decrease enterobacteria in the intestine [160]. Several studies have also shown that protein hydrolysates from pulses possess angiotensin I converting enzyme ANFs inhibitory properties and have bile acid binding properties and may help to prevent cardiovascular disease [159,160]. In general, they are extremely important in human diet due to their anti-carcinogenic, anti-inflammatory, antimutagenic, hypoglycaemic, immunomodulatory and neuroprotective attributes [159,160]. Anti-nutritional compounds are generally reduced through various processing methods, such as dehulling, soaking, roasting, germination, baking, boiling, steaming, autoclaving, extrusion cooking, fermentation, gamma irradiation, and other treatments [2,14,15,31]. These processes help to enhancing protein digestibility, nutritional, sensory, and functional properties, and preserve protein content [15,27]. The germinated legume seeds have been shown to have fewer antinutritional substances, such as enzyme inhibitors, phytates, condensed tannins, glycosides, vicine, and convicine [50]. Importantly, raffinose family oligosaccharides causing flatulence and thus reducing the utilisation of pulses are shown to be degraded during germination [50]. Fermentation as a bioprocessing tool has also been shown to impact the chemical composition of pulses, for example, by reducing the amount of ROF and altering the phenolic and protein profiles [50]. Furthermore, breeding strategies have led to the development of faba bean varieties with reduced levels of anti-nutrients. New low-vicine and low-tannin cultivars of faba beans have been developed to further improve their nutritional value and safety [8,21].

### 8.2. Volatile Odorant Compounds

Volatile compounds are organic molecules with a low molecular weight (<300 Daltons) and strong hydrophobicity. Because of their high volatility, they can reach the olfactory receptors. They include aldehydes, sulphur-containing compounds, alcohols, ketones, carboxylic acid, methoxypyrazines, and terpenes [8]. Volatile odorant compounds are a key factor influencing the aroma and overall sensory perception of pulse proteins, which can significantly impact their acceptance in food applications [37,162]. Volatile generation starts throughout seed development and changes as the seed is harvested, processed, and stored. When pulses are exposed to extreme adverse conditions such as temperature, mechanical damage, insect infestation, and water stress, the production of volatiles is typically increased. Their synthesis is hence frequently a defensive reaction to adverse situations [8]. The undesirable aroma of legumes is due to their emission of volatiles developing from the fatty acid oxidation catalysed by lipoxygense. Aldehydes, alcohols, ketones, acids, and pyrazines are the primary contributors to the musty, beany, grassy, earthy, and leafy unwanted odors in faba bean seeds [17,162]. These volatile compounds are produced during processing through Maillard reactions, enzymatic activities, and lipid oxidation [37]. *Vicia faba* L. *minor* has many interests but is characterised by off-notes (negative odours/aromas) due to volatile compounds that are promoted during seed processing. Little is known about the volatile compounds of faba beans and their contribution to its odour [36]. The flavour characteristics of faba beans are generally affected by type of cultivar, storage conditions, processing, and geographical location [8].

### 8.3. Non-Volatile Taste Compounds

Taste and aroma metabolites, which are frequently non-volatile, contribute to flavour profiles by highlighting volatile aroma metabolites, which in turn improve the gustatory experience. Five categories are used to classify taste experiences: umami, bitter, sour, salty, and sweet. Tasting plant-based products involves a variety of chemical metabolites [8]. In order to perceive the flavour of faba beans, volatile odorant compounds and non-volatile taste metabolites both of which are classified under separate chemical classes interact. Therefore, in faba bean ingredients, both volatile and non-volatile compounds affect the entire perceived flavour response. Certain glycosides, peptides, tannins, alkaloids, and polyphenols are frequently linked to bitterness in faba bean elements. A significant amount of these metabolites can be found in raw faba bean [8]. Bitterness in pulses arises from a variety of compounds, with research highlighting the roles of lipids, lipid oxidation products, peptides, saponins, and phenolic compounds. However, most studies have focused on peas or soybeans, leaving faba beans less explored in this context [17]. The primary phenolic substances found in faba bean include phenolic acids, flavonols, flavanols, flavanonols, flavanones, and isoflavones. Polyphenols are secondary metabolites associated with bitterness, especially for the flavonoids class, although they have their associated health benefits related to anti-inflammatory function and other health benefits. Other non-volatiles metabolites include hydroperoxy epoxides, hydroperoxy cyclic peroxides, and dihydroperoxides [8]. Saponins give faba bean ingredients a bitter, metallic off-flavour. The majority of saponins might be reduced primarily in concentrate and isolate because they are typically heat stable and hence challenging to remove during processing. These non-volatile compounds typically bind to proteins, which makes them more stable [8]. Recent findings suggest that the concentration of saponins in faba beans is too low to contribute significantly to their bitterness. Many alkaloids, such as caffeine or quinine, are known to contribute to food product bitterness. Two alkaloids, vicine and convicine, have been identified in faba beans [17]. Instead, vicine, a major alkaloid in faba beans, has been shown to activate one of the 25 human bitter taste receptors (TAS2R), suggesting that vicine plays a partial role in the bitterness of faba beans [37].

### 8.4. Off-Flavour

Pulses are characterised by unpleasant flavours (off-flavours) which limit consumer acceptability. These off-flavours consist of unpleasant notes (off-notes), bitter taste (off-taste) and astringency. Faba beans are not astringent like peas and common beans, which supports their sensory interests. Research on pulses and pulse-based products has largely focused on the involvement of volatile compounds in the off-notes [17]. *Vicia faba* L. *minor* is gaining attention as a sustainable plant-based ingredient due to its environmental, nutritional, and functional benefits. However, its consumption is limited by off-flavours/off-notes (negative odours/aromas), including bitterness and unpleasant aromas, attributed to volatile compounds produced during seed processing [36]. Although little is known about the specific volatile compounds in faba beans and their exact contributions to its aroma, research suggests that *Vicia faba* L. *minor* exhibits milder off-notes and less astringency compared to other pulses such as peas and soybeans. This makes faba beans more appealing from a sensory perspective despite their inherent off-flavours [17]. Research indicates that compounds such as saponins, phenolic compounds, peptides, lipids, and lipid oxidation products contribute to bitterness in pulses [17]. The off-flavours in faba bean seeds were released during the harvest, processing, and storage time [162,163]. In faba beans, bitterness is mainly associated with alkaloids like vicine and convicine, though saponins and tannins play a minor role [17,36]. Vicine, in particular, activates human bitter taste receptors (TAS2R), partially explaining the beans bitterness. Off-flavours in faba beans include both non-volatile compounds (causing bitterness and astringency) and volatile compounds (producing off-notes like grass, metallic, rancid, or yeast-like odors) [36,37].

Despite its benefits, faba beans are primarily used in animal feed due to the presence of these off-flavours [36,37]. The unpleasant characteristics (off-flavours) include dried pea, fresh pea, grass, metallic, mouldy, rancid, and yeast notes [28]. However, since they lack the astringency of peas and common beans, faba beans remain of sensory interest for food applications [28]. Despite the growing interest in pea protein ingredients, their inclusion in foods and beverages remains a significant challenge for the food industry. This difficulty is primarily due to the inherent beany flavour of pea proteins and their impact on functional and technological properties [14]. The presence of off-flavour compounds, including beany and green notes, is closely linked to the natural occurrence of aldehydes, ketones, furans, pyrazines, and alcohols in peas. These compounds contribute to sensory perceptions such as “green”, “grassy”, “hay-like”, and “pea pod” aromas [14]. During dry or wet pea protein processing, these off-flavour compounds tend to bind to pea proteins. Techniques such as fermentation (using bacteria, yeast, or fungi), enzymatic treatments, and chemical or thermal processing can modify protein structures to reduce the number of accessible binding sites. This decreases protein-flavour binding affinities, thereby improving sensory perception [14]. Moreover, volatile compounds responsible for off-flavours (e.g., alcohols, aldehydes, ketones) can result from lipid oxidation during germination, harvest, storage, or processing. This not only impacts flavour but also leads to a loss of essential amino acids and protein functionality [14]. Both conventional and innovative processing techniques are being explored to mitigate undesirable flavours and improve the technological and physiological performance of pea protein to meet industry and consumer expectations [4,14]. Technologies and methods such as control of oxidation, germination, dehulling, enzymatic treatment, heat processing, fermentation, milling, and cultivar selection have been described to prevent the off-flavours development and accumulation in pulses [162].

### 8.5. Protein Bioactivity and Allergenicity

Pulses are an affordable and environmentally responsible source of biologically active proteins and peptides with antimicrobial and anti-oxidant properties [26]. Bioactive peptides are brief peptides with 2 to 20 amino acids that are either released during protein hydrolysis or naturally found in the food matrix. After the protein is hydrolysed, those amino acid sequences that were not specifically active when contained in the original protein structure become extremely active. Peptides are thought to have a wide variety of bioactivities, including antimicrobial, anticancer, anti-inflammatory, anti-hypertensive, anti-diabetic, ACE inhibitory, cholesterol-lowering, opioid, and antioxidant properties [21,76]. The antioxidative effects are the result of scavenging or absorbing free radicals and reactive oxygen species, which depend on the hydrophobicity, molecular mass, and amino acid composition/sequence, as well as the protein source, hydrolysing enzyme, and hydrolysis conditions [21,26]. The antimicrobial activity of pulse proteins/peptides is attributed to their capacity to interact with components of bacterial/fungal cells or the viral envelope [26]. Hydrolysates of the protein from faba beans have been made under non-physiological conditions to provide novel biofunctional food elements. A commercial pulse flour mixture (pea, lentil, and faba bean) hydrolysed by a protease mixture frequently used in baking industry yielded a small number of faba bean peptides having fungicide properties [21].

In addition to the bioactivities mentioned above, legume-derived protein hydrolysates also had other activities, including immunomodulatory activity, hypocholesterolemic activity, and antigastrointestinal cancer, among others [76]. Faba bean protein hydrolysate can help maintain the health of skeletal muscles by encouraging the synthesis of skeletal proteins and preventing the loss of muscle due to persistent inflammation. Participating in these activities could help prevent sarcopenia as people age, among other things [21]. Remarkably, the only hydrolysate that had the ability to promote protein synthesis was the faba bean hydrolysate. In a pre-clinical investigation, the efficacy of this unique functional component was confirmed both in vivo and in vitro using cell models. Using a predictive machine learning technique, the peptide profile of this intricate functional food ingredient was examined for anti-inflammatory and protein synthesis-promoting properties [21]. Faba bean protein hydrolysates had strong hypocholesterolemic activity, mainly by blocking bile acid or cholesterol absorption, inhibiting cholesterol synthesis, and stimulating low-density lipoprotein (LDL) receptor transcription, to reduce the level of blood cholesterol [21]. Protein hydrolysates have significant anticancer activities because they contain amino acids that could interact with adhesion factors, including arginine, aromatic amino acids, and glutamine. In addition, legume protein hydrolysates could play an anti-proliferative role in gastrointestinal cancer cells by reducing mitochondrial membrane potential and promoting DNA damage [76]. Faba bean proteins may cause hypersensitivity reactions because they belong to the legume family [21]. Lentils, peas, and beans are among the other legumes that have been found to contain several allergies. There is, however, a lack of information regarding faba bean allergens. According to a recent study, faba beans had one of the lowest prevalences of sensitisation to different legumes among 106 randomly selected allergic patients [21]. According to research, a skin prick test (SPT) and specific IgE-binding protein identification in a few instances confirmed allergic specific reactions to faba bean proteins, indicating that faba beans may contain a variety of allergenic proteins and peptides [21]. Pea protein is not commonly considered as a allergenic food or ingredient, however, its allergic reactions have been reported as pea protein or pea protein isolates has become more prevalent in the food marketplace [53]. Several allergens, such as Pis s 1 (50 kDa vicilin), Pis s 2 (64 kDa convicilin), Pis s 5 (profilin), Pis s 6 (17 kDa PR10 protein), Pis s albumin (26 kDa) and an agglutinin (30 kDa), have been identified from pea protein [53]. Pis s 1, one of the major allergens in field pea, has a 60% to 65% homologous sequence with peanut Ara h 1 allergen. The sequence similarity of glycinins among peanut, soybean and pea is in the range of 62–72% [53]. Allergenicity induced by different legume proteins demonstrated high degree of immunological cross reactivity because legumes have structurally homologous proteins and share common epitopes [53]. However, a low cross reactivity has been reported between pea globulins and peanut proteins [53].

### 8.6. Technologies Used to Reduce Allergic Proteins

Legumes can be made safe for human consumption by carefully processing them to remove anti-nutrient components [120]. Food allergies are one of the main challenges to the widespread use of plant-based foods and meat substitutes. In terms of the chemical and biological alteration of the protein allergens from gluten and leguminous foods, there are still a lot of unexplored and undeveloped possibilities [154]. Germination and cotyledon removal are a useful approach to produce pea products for people who suffer from food allergic disorders [53]. Fermentation is a process that can reduce allergenicity by protein degradation, acid-induced denaturation, glycosylation, and microbial activity-driven Maillard reactions. From a more biological perspective, microbial strains and fermentation metabolites can activate Treg induction, regulate T-helper cell Th1/Th2 responses, maintain the epithelial barrier integrity, and increase gut microflora diversity, all mechanisms that contribute to reduction in allergenic reaction [154]. Treg induction, for example, inhibits autoimmune responses and increases the threshold value for an immune response. In terms of Th1 and Th2 responses, allergies have been shown to favour the latter and be negatively regulated by Th1 cells. Changes in protein size (resulting from protein fragmentation) caused by fermentation normally maintain or contribute to a decrease in IgE-binding capacity in 2 S albumins, legumins, and vicilins [154]. Fermentation is increasingly being “rediscovered” as a process due to its myriad nutritional and health effects, notably including food protein allergen degradation or transformation [154]. Enzymatic catalysis can be a type of enzymatic degradation and/or cross-linking. Enzymatic degradation or proteolysis (catalysed hydrolysis) mainly destroys the IgE-binding components to reduce allergenicity. However, protein resistance to enzymatic digestion, such as pepsin digestion, cannot be used as a good predictor of allergenicity [154]. Lipids also play a role during the digestion process, as, through their interactions with the allergenic proteins, they may stabilise them and preserve their structure-related allergenicity. Meanwhile, enzymatic cross-linking is a non-thermal process that produces high-molecular-weight polymerized allergens with reduced immunoreactivity and IgE-binding potential. Both processes can be combined to substantially reduce food protein allergenicity [154]. Microwave, high-pressure, and ultrasonication processes are some of the potential physical pretreatments that might be combined with chemical and biochemical techniques to reduce food allergenicity. Microwave energy may reduce allergenicity through: (1) protein aggregation and structural changes, (2) the destruction of allergen epitopes through subsequent enzymatic hydrolysis, and (3) the disruption of disulfide links in the protein, lowering their stability and increasing their susceptibility to the enzyme break-down [154]. Furthermore, by choosing and domesticating appropriate genotypes based on anti-nutritive compounds, it is also possible to help underutilized legumes in adapting. Advances in biotechnology have led to a considerable reduction in allergenic proteins and secondary metabolites [120]. The limitations of field pea and field bean proteins for human consumption are summarised in Table 7.

## 9. Food Application and Its Health Benefits

Dry seeds of peas have long been used globally as a staple food and feed, and its nutritional, health and ecological benefits comply with growing demand for novel vegan foods intended for health and sustainability conscious individuals [40]. Protein isolates from various vegetable crops are widely used to replace traditional raw materials in yoghurt and sausage recipes [19]. Pea protein has been widely used in a variety of food formulations of plant-based, vegan and meat-analogues products, such as bread, pasta, meat products, and beverages, to increase protein content in diets. Additionally, it serves as a binder, emulsifier, stabilizer, or extender in foods [108,122]. Pea protein serves various roles, such as a stabilizer, emulsifier, film-forming agent, and meat replacer, due to its balanced amino acid profile and functional properties [10,41] Pea protein isolate (from *Pisum sativum* var. *arvense*) is emerging as a valuable raw material in the food industry, especially to replace soy protein in meat products like sausages [19]. Their solubility and ability to form soft gels make them ideal for dairy analog drinks, fermented products, and curds [2,10,41]. Pea protein can be used as a food emulsifier, encapsulating material, and a biodegradable natural polymer [10]. In addition to traditional pea soups, casseroles, boiled and/or baked, roasted peas as staple food and humus made from canned, frozen or dried peas, there are many new vegan foods made with field pea fractions recently launched on the global food market [40]. Major novel vegan foods with pea ingredients could be categorised in such groups: meat product analogues (plant-based burgers, sausages, “meat ball alternatives”, “minced meat alternatives” etc.), dairy product alternatives (drinks, yoghurt-like gels, cheese-like alternatives, nondairy sports products), food supplements (protein powders, emulsifiers, therapeutic beverages), snacks (energy bars, extrudates), fortified cereal products (traditionally made with wheat bread, pasta, crackers) and other [9,40,53]. In addition, pea proteins have been found suitable for preparing gluten-free muffins with characteristics comparable to those made using wheat gluten [2,10,14,19,41,53].

Pea protein can also be used as nutritional supplements for sports and exercises. Leucine, isoleucine and valine are three essential branched-chain amino acids (BCAA) which have an aliphatic side chain with a branch and can promote muscle growth. The pea protein is an excellent source of BCAAs and have high and balanced contents of leucine, isoleucine and valine [53]. Similar to peas, faba beans are eaten in a variety of food products, such as stewed broad beans (Medamis), stewed broad bean cakes (Taamia), dried seeds, frozen or canned snacks, and stewed broad bean paste (Bissara). They are also used to prepare thick gruels, purees, and soups made with germinated broad beans. Furthermore, faba beans are frequently eaten for breakfast in pastes, stews, and soups. Fresh green pods, on the other hand, are usually cooked and eaten as vegetables. In addition, many developed nations use faba beans extensively as animal feed for horses, pigs, and poultry [30]. Faba bean protein isolate is gaining significant attention in the food industry for its nutritional and functional properties. It serves as an alternative to traditional ingredients like wheat flour, casein, and whey in various food products, including sausages, beverages, and meat analogues [27]. Additionally, the protein isolate is a popular choice in gluten-free foods such as breads, snacks, and pasta, catering to health-conscious and gluten-sensitive consumers [2]. The adoption of plant-based diets has long been linked to a healthier lifestyle and a reduced risk of diseases such as type 2 diabetes (T2D), obesity, cancer, and coronary heart disease, which remains the leading global cause of death [2]. In this context, faba beans stand out for their nutrient-rich profile, offering potential medicinal benefits such as improved heart health, blood sugar regulation, weight management, and anti-inflammatory effects. In addition, faba beans are beneficial for bone health, digestive wellness, and as a significant source of folate-important for prenatal care [2]. Faba bean protein is rich in phenolic compounds, particularly polyphenols, which have antioxidant, anticarcinogenic, and antimutagenic properties. These antioxidants not only provide health benefits but also extend the shelf life of food and feed products. Due to these nutritional benefits, pea and faba bean protein isolates are increasingly being applied in food formulations to enhance nutritional profiles and meet the demand for healthier, gluten-free and protein-enriched products [27,35].

Consuming dry seeds and pea protein on a regular basis may help lower the risk of diet-related diseases such as cardiovascular disease, heart disease, diabetes, obesity, high blood pressure, and some types of cancer, such as colon, breast, and kidney cancer [40,53]. According to Rasskazova at al. [40], this effect has been directly linked to their low glycaemic index and chemical content. They have also traditionally been used as a vegan and vegetarian protein substitute. Pea proteins have anti-inflammatory, anti-hypertensive, antioxidant, and cholesterol-lowering properties in addition to being hypoallergenic. Also, they contain bioactive peptides that support cardiovascular health by inhibiting the angiotensin I-converting enzyme and offering antioxidant activity. Pea protein is becoming more and more popular in functional food since it may also reduce appetite by controlling hormones linked to hunger, glucose absorption, and stomach emptying [10]. In general, pulse-based protein isolates and ingredients are used in innovative foods such bakery products, meat analogues, dairy alternatives, pasta, and beverages. Beyond these uses, new uses for pulse proteins must be investigated in infant and child formula, beverages, breakfast cereals, flavour development, and extruded snack products. Additionally, new applications for pulse proteins are required, such as personalized and precision nutrition and bioactive peptides, and innovative technologies including 3D printing, extrusion, and artificial intelligence must be used for pulse protein research [166].

## 10. Concluding Remarks and Future Research Directions

The review highlights the significance of underutilised legumes (fodder seeds) in human and animal diets. These legumes provide essential nutrients and can adapt to diverse climatic conditions, soil health through nitrogen fixation and environmental advantages. The underutilised legumes are a rich source of proteins, vitamins, minerals, lipids, fibre, and carbohydrates, they offer a balanced diet. Moreover, the nutritional and anti-nutritional contents of these legumes help in solving the health related issues by supplying essential nutrients to humans through alternate yet necessary food sources. However, their limited use is attributed to insufficient knowledge, research funding, and academic interest. The current agricultural system focuses on a few mainstream crops, despite these contributing to food security efforts. Utilising non-traditional food sources like underutilised legumes is essential in addressing global hunger and nutrition challenges. Additionally, while faba bean and pea derivatives are underused, they have the potential to serve as meat and dairy protein substitutes or functional food additives, similar to soy protein.

## Figures and Tables

**Table 1:** Proximate composition (%) of *Vicia faba* var. *minor* and *Pisum sativum* var. *arvense* seeds reported by various authors ^a^.

Pulse	Protein	Ash	Fat	Fibre	Carbohydrates	Energy (kJ/100 g)	Moisture	References
Field bean	22.7–28.3	2.72–3.41	1.0–2.0	11.37–16.59	NR	NR	NR	[24]
	30.4–31.6	NR	NR	NR	51.3–51.9	NR	NR	[47]
	25.78–29.13	NR	2.52–2.61	NR	NR	NR	NR	[36]
	25.4–26.8	3.3–3.6	1.1–1.3	7.9–7.5	41.0–41.3	1610.0–1620.0	NR	[49]
	35.3	NR	NR	NR	43.2	NR	NR	[50]
	29.63	2.90	1.06	NR	NR	451.0	NR	[51]
Field pea	19.21	3.41	1.61	NR	NR	NR	75.60	[19] ^b^
	16.14–20.32	2.86–3.22	0.90–2.17	1.56–3.39	58.46–64.08	1376.5–1418.4	12.31–13.44	[52]
	23.0	3.0	4.0	NR	56.0	NR	NR	[44]
	20.0–25.0	NR	NR	10.0–20.0	40.0–50.0	NR	NR	[53]
	20.5–22.6	3.2	2.0–3.0	2.5	17.0–22.0	NR	NR	[54]
	20.37	2.3	1.57	8.72	51.16	NR	NR	[55]
	21.0–24.0	1.9–2.2	1.5–2.0	NR	42.0–46.0	NR	10.0–15.0	[56]

^a^ Results are expressed on a dry weight basis. ^b^ Results are expressed on a wet weight basis. NR—Not Reported.

**Table 2:** Mineral composition and Vitamin content (mg/100 g) of *Vicia faba* var. *minor* and *Pisum sativum* var. *arvense* seed reported by various authors ^a^.

	Field Bean			Field Pea	
	References				
Minerals and Vitamins	[24]	[49]	[51]	[19]	[13] ^b^
Ca	NR	140	72.86	26	NR
Fe	1.8–21.3	5.9–7.3	8.92	1.57	NR
Zn	0.9–5.2	3.1	5.28	1.54	NR
Mg	NR	160.0–170.0	142.63	35	NR
Mn	NR	0.7	1.59	0.46	NR
K	NR	980.0–1000.0	1548.61	255	NR
P	NR	460.0–470.0	654.55	110	NR
Cu	NR	1.1–1.2	NR	0.196	NR
Vitamin C	NR	NR	NR	NR	43.82
Vitamin E	NR	0.5	NR	NR	NR
Vitamin B1	NR	0.5	NR	NR	NR
Vitamin B2	NR	0.3	NR	NR	NR
Niacin	NR	2.5	NR	NR	NR
Pantothenic acid	NR	0.3	NR	NR	NR
Choline	NR	$1.7\cdot 10^{6}$	NR	NR	NR

^a^ Results are expressed on a dry weight basis. ^b^ Results are expressed on a wet weight basis. NR—Not Reported.

**Table 4:** Overview of Pulse Protein Extraction Methods: Advantages, Limitations, Techno-Functional Properties, Applications, and Future Research Trends.

Extraction Methods	Advantages	Limitations	Effect on Techno-Functional Properties	Nutritional Value	Applications in Food Industry	Future Perspectives & Research Trends	Examples	References
Dry Fractionation (Air Classification & Size Reduction)	Maintains native protein functionality. Energy-efficient, chemical-free treatment. Clean-label process.	Lower protein purity (50–60%). Limited protein solubility and functionality. Off-flavours and relatively high level of antinutritional factors. Particle-particle collisions may happen.	Higher emulsification and foaming and water-holding capacity. Low solubility.	Preserves bioactive compounds, micronutrients and fibre content. Moderate protein yield.	Pea protein concentrates. Protein-enriched bakery and snack products.	Improving solubility through milling/thermal treatment. Exploring hybrid methods combining air classification/electrostatic, or dry/wet techniques	Common for producing protein concentrates. Pea and faba bean protein concentrate, lentil protein flour	[10,14,41,79,80]
Wet Fractionation (Alkaline Extraction/Isoelectric Precipitation—AEIEP)	High protein purity (up to 90%) and high yield. Little off-flavours and a low level of antinutritional factors. Low fat content.	Intensive use of energy and water. Chemical use may denature proteins or affect native functionality. Loss of micronutirnts.	High solubility at specific pH levels. Improved emulsifying and gelling properties.	High protein content but potential loss of heat-sensitive nutrients (e.g., vitamins) High digestibility and bioavailability	Plant-based protein isolates for dairy/meat alternatives. Beverages, protein bars.	Reducing chemical use, optimizing protein extraction yield. Researching cost-effective, eco-friendly extraction methods.	Common for producing protein isolates. Fava bean, pea and soy protein isolate	[10,41,75,78,79]
Salt Extraction	Produces high-purity protein (80–90%). Reduce processing time and cost. Reduce solvent consumption	Aggregation of protein may occur. Low protein extraction rate and purity. Water-intensive and high waste streams.	High solubility and binding properties of proteins to improve texture. Increasing hydration and water oil binding and foaming capacity.	Retains essential amino acids but may lose minerals due to dialysis.	Functional foods and beverages, and specialized food products with high bioactivity.	Research into cost-effective and scalable salt extraction methods. Exploring protein-specific optimization.	Pea, fava bean, chickpea, and lentil protein isolate.	[10,41,83,88]
Ultrafiltration	Relatively simple and produce high purity and quality (90–95%). Maintains native protein structure. Minimal product degradation.	Fouling and concentration polarization challenges occur and cause low efficiency. Time-consuming and high operational cost. High level of antinutritional factors.	High solubility, emulsifying, foaming and gelling functionality. Improved water absorption and oil binding capacity.	Retains bioactive peptides and functional proteins.	High-functional protein products for beverages and protein supplements.	Researching to address fouling and concentration polarization challenges.	Pea, chickpea and lentil protein isolates.	[41,76,83]
Ultrasound-Assisted Extraction	Enhances protein yield and extraction efficiency. Reduces extraction time and energy.	Expensive equipment. Potential for protein denaturation and reduce the protein concentration if conditions are not optimized.	Enhances solubility, emulsification, foaming and oil holding capacity. Shortens extraction time.	Improves digestibility and reduces anti-nutritional factors.	Applied for protein enrichment in beverages and bakery products.	Scaling up and optimizing ultrasound settings for improved yield. Research on protein bioactivity preservation.	Ultrasound-extracted pea and faba bean proteins.	[68,98,99]
Enzyme-Assisted Extraction	Increases the protein yield and purity. Low energy consumption and decreased waste formation. Reduces anti-nutritional factors.	High enzyme costs. Risk of incomplete protein extraction if not optimized.	Improves solubility and emulsification.	Enhances protein digestibility. Increases bioactive peptides.	Functional protein ingredients for nutraceutical and functional food products.	Exploring enzyme blends to maximize extraction efficiency. Optimizing conditions to enhance protein functionality.	Lupin and soybean protein extraction.	[68,98,102]

**Table 5:** Amino acid and fatty acid profile (mg/g) of *Vicia faba* var. *minor* and *Pisum sativum* var. *arvense* seed reported by various authors ^a^.

	Field Bean		Field Pea	
	References			
Essential Amino Acids	[24]	[49]	[19]	[53]
Lysine	44.8–74.8	16.6–17.2	3.2	47
Threonine	26.6–38.0	9.1–9.5	2.2	25
Leucine	50.8–72.1	19.3–20.3	3.3	57
Isoleucine	20.7–33.1	10.3–10.9	2	23
Methionine	NR	1.8–1.9	0.9	3
Phenylalanine	23.0–36.6	10.7–11.2	2.1	37
Valine	32.9–42.3	11.5–12.1	2.4	NR
Histidine	13.3–35.9	6.4–6.8	1.1	16
Non-Essential Amino Acids				
Alanine	45.6–57.9	10.5–11.0	0.24	NR
Asparginine	84.1–120.5	30.4–32.2	NR	NR
Aspartic Acid	86.4–109.5	NR	5.00	NR
Glutamic Acid	125.6–187.9	44.8–47.2	7.5	NR
Arginine	52.1–121.0	24.1–26.1	4.3	NR
Proline	32.9–48.2	10.5–11.1	1.8	NR
Serine	39.5–52.1	12.9–13.6	1.9	NR
Tyrosine	18.6–41.5	8.1–8.5	1.5	NR
Fatty Acids (mg/g)	[36]	[49]		
Palmitic acid C16:0	NR	1.5–1.8		
Stearic acid C18:0	NR	0.2–0.3		
Oleic d	3.91–4.52	2.3–2.8		
Omega-3 (α-Linolenic acid)	7.31–9.02	4.4–5.3		
Omega-6 (Linoleic acid)	0.41–0.58	0.3–0.4		

^a^ Results are expressed on a dry weight basis. NR—Not Reported.

**Table 6:** Provides the possible modification techniques for pulse proteins, highlighting their advantages, limitations, effects on techno-functional properties.

Modification Technique	Advantages	Limitations	Effect on Techno-Functional Properties	Examples	References
Physical Modification					
Thermal Treatment	Denatures proteins, improves digestibility and reduces anti-nutritional factors	May cause loss of sensitive amino acids and bioactive compounds	Enhances emulsification, solubility, water-holding capacity, foaming and gelling	Heating of faba bean, kidney bean and pea proteins	[76,77,125,130]
Extrusion	Reduce heat-labile ANFs, increase protein and starch digestibility	Nutritional loss	Enhances gelation and water-holding capacity	Pea, faba bean, and soy protein, chickpea flour	[31,66,90,126,139]
High-pressure Processing	Enhances protein solubility, emulsification, and gelation	Expensive and may not eliminate all anti-nutritional factors	Improves solubility, gel formation, foaming and protein structure	HPP-treated fava bean, lentil protein	[21,103,130]
Ultrasonication	Reduces particle size, improves solubility and foaming ability	Limited impact on anti-nutritional compounds, high-energy consumption	Enhances emulsification, foaming, and solubility	Ultrasound-treated faba bean, lentil protein	[2,21,24,43]
Cold plasma	Microbial inactivation, quick processing and no thermal damage	May affect the overall flavour and colour	Enhances solubility, gelling properties and water-binding capacity	CP-modified pea protein	[22,90,119]
Chemical Modification					
pH shifting treatment	Solubilizes proteins, improves emulsifying properties	Degrades amino acids, may create off-fflavours, and reduce nutritional quality	Alters solubility, emulsion stability, foaming, and protein structure	Alkali-modified pea and soy proteins	[2,47,71,127]
Glycation (Maillard Reaction)	Minimize adverse effects on flavour and colour, improves digestibility and inactivate enzyme inhibitors	Potential to form harmful advanced glycation end products	Improves solubility, emulsification, and thermal stability	Glycated pea, soy and yellow pea proteins	[110,127]
Phosphorylation	Improves digestibility	Less desirable for food applications, possibility of toxicity of residues	Improves emulsifying, solubility, gelling, foaming, and oil absorption ability	Phosphorylated pea and soy protein isolate	[90,127,145]
Acetylation and Succinylation	Improves water solubility, emulsifying properties.	Chemical reagents may leave residues and alter protein structure	Alters surface properties, increasing solubility and functional properties like foaming and emulsifying ability	Applied in mung bean, pea and faba bean proteins	[90,110]
Deamidation	Enhance the nutritional quality and sensory properties	Chemical reagents may leave residues	Improves solubility, emulsifying capacity	Deamidated pea and soy protein	[10,90,110]
Biological Modification					
Fermentation	Increase bioavailability, producing bioactive peptides, enhances flavour, reduces anti-nutritional compounds	Time-consuming and requires specialized microbes	Improves solubility, oil-holding capacity, and foaming properties	Fermented lupin, pea, and faba bean proteins	[90,153,154]
Enzyme treatment	Reduce anti-nutritional factors like off-flavours, improves nutritional value and sensory properties, and produce bioactive peptides	Complex processing, high enzyme and energy cost	Improves solubility, emulsification, oil absorption, foaming and bioactive peptide content	Enzyme treated pea and faba bean proteins	[5,9,69,121]
Germination, Sprouting	Reduces anti-nutritional factors, enhances digestibility and improves nutritional value	Limited large-scale applicability, variability in results	Enhances water-binding capacity, solubility, foam stability and gelling	Germinated chickpea, faba beans and lentils	[69,156]

**Table 7:** Summarizing the limitations for human consumption of field pea and field bean proteins.

Limitation	Field Pea	Field Bean	Details	References
Antinutritional factors	Contains phytates, tannins, trypsin inhibitors, oxalates, and saponins, lectins, and etc.	Contains tannins, lectins, trypsin inhibitors, vicine and convicine, which can cause favism (especially in individuals with G6PD deficiency).	These compounds can reduce the bioavailability of iron, calcium, and other essential nutrients and protein digestion.	[12,14,21,75,107]
Allergenic potential	Low but possible allergenic potential, particularly in individuals sensitive to legume proteins.	Potential allergenicity, especially in individuals with legume allergies or faba bean sensitivities.	Field pea proteins have a lower allergenic risk compared to other legumes, but may still trigger reactions.	[9,21,93]
Digestibility	Moderate digestibility due to the presence of fibres and antinutritional factors.	Lower digestibility because of high levels of fibres, tannins, and lectins.	Pea proteins may cause bloating and indigestion due to fibre content.	[21,53,77]
Flavour and sensory issues	Grassy, beany flavours may be undesirable in certain food applications.	Strong bitter and astringent flavours limit consumer acceptability	Both proteins require flavour masking or removal strategies in processed foods.	[14,17,36]
Gastrointestinal effects	High fibre content can lead to flatulence, bloating, and gas formation.	Can cause gastrointestinal discomfort, such as bloating, especially in sensitive individuals.	Enzyme treatments or fermentation can help reduce these effects.	[8,9,15,24,75]
Vicine and convicine (Favism risk)	Not present in field pea, so no risk of causing favism.	Contains vicine and convicine, which can trigger favism in genetically predisposed individuals.	Favism occurs due to the consumption of fava beans by individuals with G6PD deficiency.	[16,21,164,165]
Heat-induced changes	Proteins may undergo denaturation during high-temperature processing, reducing solubility and functionality.	Field bean proteins may lose functionality under excessive heat, affecting food texture.	Both proteins are heat-sensitive and may require mild heat processing to retain functionality.	[27,76,130]
